# Engineering the Thermal and Energy-Storage Properties in Quantum Dots Using Dominant Faceting: The Case Study of Silicon

**DOI:** 10.1021/acsnano.4c11376

**Published:** 2025-01-06

**Authors:** Pavel Galář, Jakub Kopenec, Robert Král, Filip Matějka, Petra Zemenová, Milan Dopita, Prokop Hapala, Dirk König, Pavel Vrbka, Kateřina Kůsová

**Affiliations:** † Institute of Physics of the CAS, v.v.i., Cukrovarnická 10, 162 00 Prague 6, Czechia; ‡ Faculty of Mathematics and Physics, Charles University, Ke Karlovu 3, 121 16 Praha 2, Czechia; § Integrated Materials Design Lab, The Australian National University, Canberra, ACT 2601, Australia; ∥ Department of Applied Mathematics, Research School of Physics, The Australian National University, Canberra, ACT 2601, Australia; ⊥ Institute of Semiconductor Electronics (IHT), RWTH Aachen University, 52074 Aachen, Germany; # University of Chemistry and Technology, Technická 5,166 28 Praha 6, Czechia

**Keywords:** quantum dots, dominant faceting, thermal oxidation, energy storage, surface free energy, silicon, ignition

## Abstract

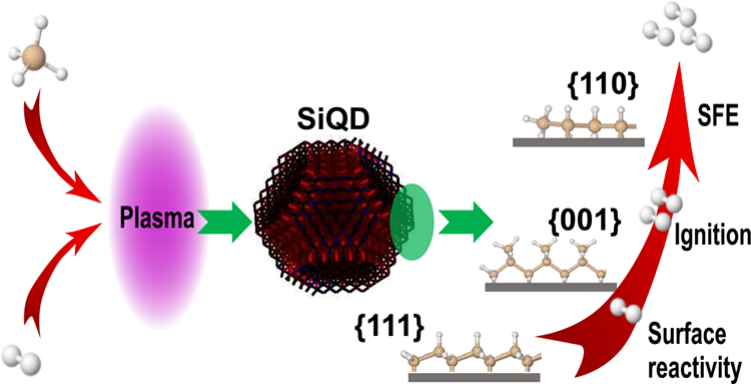

The storage and release of energy is an economic cornerstone. In quantum dots (QDs), energy storage is mostly governed by their surfaces, in particular by surface chemistry and faceting. The impact of surface free energy (SFE) through surface faceting has already been studied in QDs. Here, we introduce dominant faceting representing the structural order of the surface. In particular, we propose that realistic QDs attain complicated polyhedral quasi-spherical shapes while keeping the dominance of a certain type of facet. The type of dominant facet determines the rates of surface-related processes. Therefore, by connecting dominant faceting with SFE, trends analogical to bulk material are kept despite the lack of evident microscopic shape control. To demonstrate the applicability of dominant faceting, we synthesize sets of silicon QDs with sizes around 5 nm and classify them based on increasing SFE of the corresponding analytic geometrical models, using a detailed surface chemistry analysis. Total energies released during oxidation of the synthesized QDs reach the theoretical limit, unlike in the reference, “large” (>100 nm) silicon nanoparticles, which release about 15% less energy. Next, we perform a comprehensive experimental study of dehydrogenation and thermal oxidation of the synthesized QDs in the temperature range of 25–1100 °C, identifying SFE as the key factor determining their thermal stability and surface reactivity. In particular, four distinctive stages of energy release were observed with onset temperatures ranging between 140 and 250 °C, ≈500 and 650–700 °C, respectively, for the SFE-differing samples. Finally, the thermal oxidation of the synthesized QDs is completed at lower temperatures with increasing SFE, decreasing from 1065 to 970 °C and being > 150 °C lower in QDs than in the larger reference nanoparticles. Therefore, despite a rich mixture of features, our description based on linking dominant faceting with SFE allows us to fully explain all the observed trends, demonstrating both the potential of SFE-based engineering of energy-storage properties in QDs and the prospects of silicon QDs as an energy-storage material.

## Introduction

Diversification of ways to produce, store and reuse energy is an important task which will to a large degree influence future advancements in technology and society. Many ways to store energy so that it could be used later are being explored,^1,2^ including improvements of existing Li-ion batteries, generation of hydrogen in combination with the fuel cell technology, ground thermal storage, capacitors, flywheel energy storage, and many more. In addition to energy storage, closely related energetic materials including thermites, which rely on highly controlled release of energy, also deserve close attention.^3^ Similar to other applications, the specific properties of quantum dots (QDs) or nanostructured materials in general are employed,^4,5^ as these materials allow for tuning of a wide range of their physical and chemical properties, thus tailoring them for a specific application.

Key properties for tuning the behavior of QDs are their size, surface ligands or shell and the degree of structural order in their core. A degree of freedom which is much less explored here is the surface free energy (SFE), or in other words, the excess energy that the surface has compared to the bulk of the material. The surface of any QD must have undergone surface reconstruction to minimize its total energy, exposing different surface facets. While QDs with varied surface facets differ in SFE and thus in their stability, they also favor certain types of chemical reactions, as such chemical reactions proceed on surfaces with different orientations at substantially different rates. In ligand-passivated QDs, different surface facets also support different types of ligands, as due to geometrical constraints, each surface atoms is bonded to a given number of neighboring atoms, which leaves a certain number of bond(s) exposed.^6^ Thus, SFE is an important parameter for tailoring surface reactivity and possibly other properties of QDs.

Generally speaking, surface faceting is known to impact stability.^7,8^ As such, tuning of SFE is not an unexplored topic even in QDs. In the past, chemical or physical properties of QDs synthesized using wet-chemistry methods were linked to the geometry of model QDs. For example, in II–VI QDs, a clear connection between surface geometry and surface traps has been reported based on calculations.^9^ In lead chalcogenide PbS QDs, a size-related threshold between mixed {111} and {100} faceting and exclusive {111} faceting has been identified as the root cause of size-dependent air stability, as the {100} facets are more prone to oxidation.^10^ The synthesis of exclusively {111} faceted tetrahedral InP QDs imparted optical properties different from their spherical counterparts.^11,12^

An ideal material for the study of the influence of SFE on the energetic and energy-storage properties of QDs is silicon. There are many reasons for this choice: (i) Crystalline silicon has a relatively high melting point (≈1415 °C), due to the strong covalent bonds between its atoms, implying large volumetric energy density of 75.5 kJ·cm^–3^, which is about 2.5 × higher than gasoline. The corresponding gravimetric energy density is 32.4 kJ·g^–1^.^13^ (ii) Unlike other semiconductor materials, it crystallizes only in a single type of face-centered diamond cubic structure. Its crystalline structure is highly stable in a wide range of temperatures and even under pressure.^14^ Therefore, any observed change in behavior of this material can be assigned to the influence of the surface; potential changes in the crystalline structure can be excluded as long as the material stays crystalline. (iii) Bulk Si itself is unsuitable as a carrier of energy, because the rupture of Si–Si bonds requires high temperatures,^15^ and oxidation is limited by the extremely slow diffusion of oxygen through the surface SiO_2_ layer (1  at 1000 °C).^16^ Fortunately, the high surface reactivity of nanostructured Si overcomes these limitations.^16−19^ (iv) The direct chemical toxicity of silicon and SiO_2_ are low, even in the form of QDs,^20^ and its abundance in the Earth’s crust is extremely high,^21^ providing strong motivation for using silicon as resource-efficient environmentally friendly energy-storage material. (v) Even if SiQDs are not yet readily commercially available, they can be obtained in sufficient amounts and their structural properties can be widely tuned if they are synthesized in nonthermal plasma.^4,22^

The highly covalent nature of SiQDs brings about several challenges when compared to the more traditionally studied QD materials. The reported shapes of SiQDs are, with only a few exceptions,^23−25^ almost exclusively spherical^26,27^ and the tendency of SiQDs to form purely faceted structures might be severely limited. Moreover, covalent Si QDs require higher energies for their formation and therefore cannot be easily fabricated using wet-chemistry approaches typical for other QDs. Consequently, the methods of synthesis of SiQDs do not yet reach the same high levels of elaborate control and precise shape tuning of macroscopic amounts as in more traditional QD materials.

Nevertheless, hints as to the possible immense influence of SFE in nanostructured silicon can be found in the literature, with the most obvious one being the low-temperature ignition phenomenon. In nanoporous silicon, spontaneous combustion was reported even at the temperature range as low as 4.2–90 K under special conditions,^16^ if condensed or liquid oxygen filled the pores of H-terminated porous Si. Other ignition temperatures for nanosized Si were reported to be higher, above 600 °C,^3^ but still well below the temperatures typical for effective combustion by in-diffusion of oxygen on a large scale. These findings suggest that dehydrogenation and/or oxidation of nanostructured silicon is widely tunable if suitable conditions are applied.

In this article, we introduce the term of dominant faceting, which refers to the SFE-governed tunability of surface properties without evident microscopic shape control. The apparent contradiction of facet tuning without shape control is achieved though highly polyhedral, quasi-spherical shape of the nanoparticles, in which a certain facet still spatially dominates the surface. Thus, dominant faceting signifies that the trends inferred based on simple bulk geometrical models and SFE are also observed in QDs “on average”, that is if the QDs are experimentally studied as a macroscopic ensemble. This approach is in contrast to, and in a way a generalization of, the published reports on the tuning of properties of chemically synthesized QDs using the faceting of their surface. In order to show how surface properties and dominant faceting influence SiQDs, we focus on the ignition of Si nanoparticles fabricated by standard protocols and explain the observed phenomena using a detailed study of the dehydrogenation and thermal oxidation of surface bonds in SiQDs. In these sets of experiments, we identify SFE as a key factor determining the stability and surface reactivity of the synthesized material. In addition to the introduction of dominant faceting, our results thus provide a detailed guide to the tuning of energy-storage and thermal properties of SiQDs and explore their potential as an energy-storage material.

## Results and Discussion

### Ignition Properties of Si Nanoparticles

**Figure 1:**
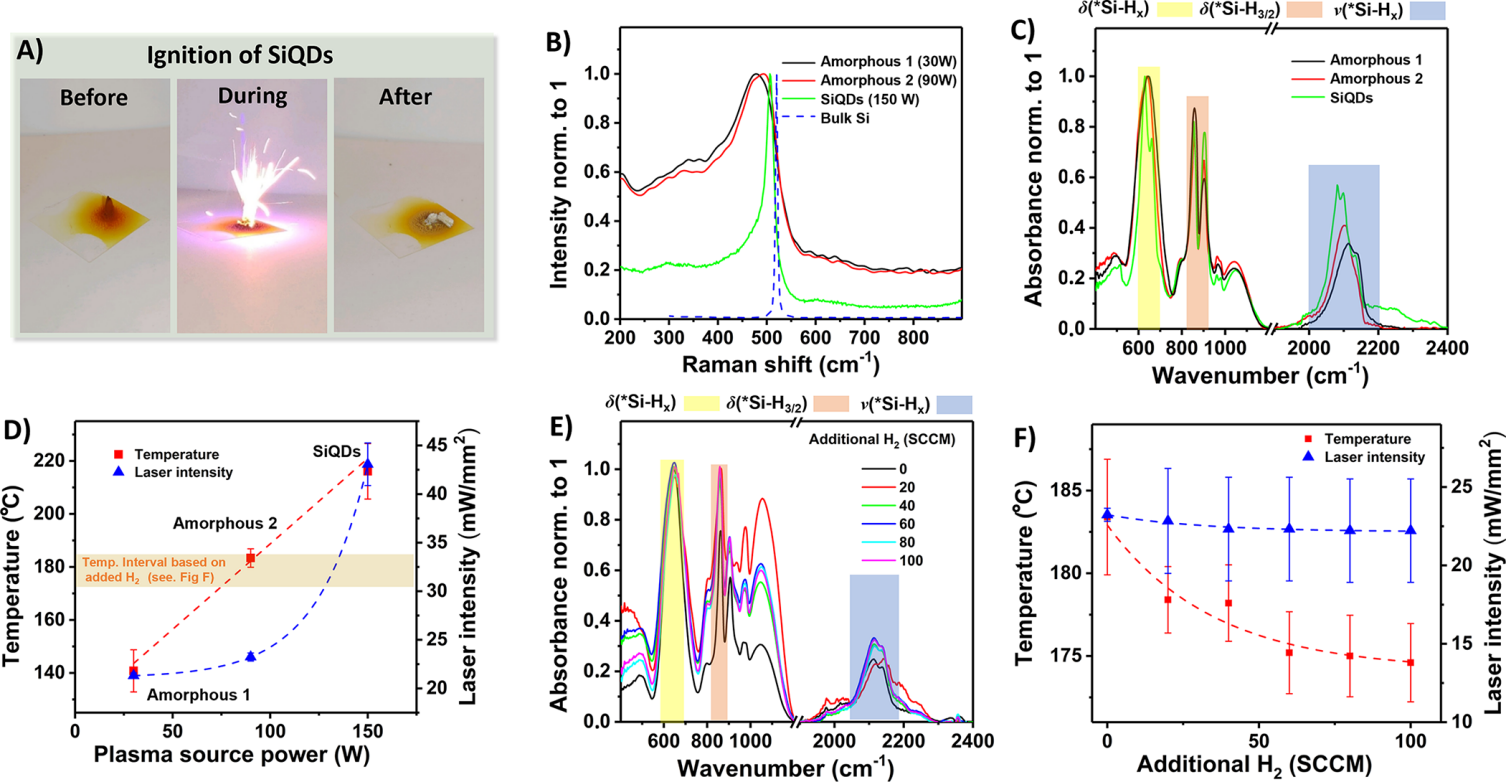
(a) Ambient-light photographs of synthesized SiQDs as deposited, ignited with a laser pointer (405 nm, 50 mW) and after combustion (from left to right). The change in color from beige-orange to white marks the complete oxidation of silicon into silicon dioxide. See a Supplementary Video File for an example of light-initiated ignition. (b) Raman spectra of Si nanoparticles fabricated with different outputs on the source, influencing the degree of structural order. Raman spectrum of bulk silicon is included for comparison as the dashed blue curve. (c) FTIR spectra showing Si–H bonds on the surface of Si nanoparticles from panel (b). (d) Ignition conditions of Si nanoparticles from panel (b). The samples were ignited on a hot plate or using a laser pointer. The dashed curves serve merely as the guides for the eye. (e) FTIR spectra of Si nanoparticles synthesized at 90 W (amorphous 2 in panel (b)) under varying flow of H_2_, effectively etching the surface of Si nanoparticles during the synthesis. (f) Ignition conditions of Si nanoparticles from panel (e). The dashed curves serve merely as the guides for the eye, and error bars are standard deviations.

In order to efficiently tune the degree of structural order of the surface, Si nanoparticles for this study were synthesized in nonthermal plasma, see Table 1 and Supporting Information Section S2.1 for details. Each sample was collected on a 150-μm-thick glass slide in the form of a cone-shape pile of lightly packed powder in the amounts of approximately 5 mg per synthesis (depending on the type of sample). Based on a simple estimate, the porosity (the volume fraction of air) in our samples^19^ is 0.77. An example of a synthesized sample is shown in Figure 1a in the leftmost photograph. In some of the synthesized samples, we observed spontaneous combustion when the sample was transferred out of the reactor and exposed to ambient conditions (not shown). The combustion of synthesized SiQDs under *controlled* conditions is shown in a Supplementary Video File and in Figure 1a. The change in the ambient-lightning color of the SiQD powder before and after the combustion from deep beige/orange to white signifies a highly efficient oxidation of the starting mostly Si-based material to SiO_2_, which was accompanied by the energy release in the form of a small explosion.

**Table 1:** List of Mean Diameters of the Investigated Samples Determined Using Different Methods, and List of the Most Important Properties of the Studied Samples and Preparation Conditions (Using 1 % Silane in Argon at 80-sccm Flow Rate as the Carrier Gas).

label		power (W)	hydrogen flow (sccm)	fresh/aged^a^	nominal size (nm)
amorphous 1		30	0	no	2−6, irregular
amorphous 2		90	0−100	no	2−6, irregular
SiQDa	{111}	150	0	yes	≈ 5−6
	{001}				≈ 5−6
	p{110}				≈ 4−5
c-SiNCs		commercial		n0	170

a The fresh/aged column shows which of the samples were studied as the two separate fresh/aged varieties (yes), and which were not (no).

To explore the combustion conditions in more detail, we fabricated three types of samples with varying input power of the source during synthesis. Power on the source predominantly tunes the structural order of atoms in the nanoparticle core, since the core crystallizes only if sufficient energy is available.^28,29^ Possibly, also the size of the produced nanoparticles can be influenced. The structural change from amorphous nanoparticles to SiQDs is documented in Figure 1b, where, at the highest power setting, the corresponding Raman spectra change from the broad peak assigned to amorphous Si to a sharper line at ≈510 cm^–1^, typical for the optical phonon in quantum-confined Si.^28,30^

The produced set of amorphous and crystalline Si nanoparticles was further characterized using FTIR measurements presented in Figure 1c. The synthesized material is mostly hydride terminated, showing only trace amounts of surface oxidation as the broad peaks at ≈1045 cm^–1^ assigned to the antisymmetric stretches of Si–O–Si.^31−33^ The typical 2000–2200 cm^–1^ region marked as ν(*Si–H_*x*_, whereby *Si stands for silicon on the surface of an Si nanoparticle), is clearly associated with the *Si–H_*x*_ stretching modes,^26,34−39^ namely with ν(*Si–H) at 2080 cm^–1^, ν(*Si–H_2_) at 2098 cm^–1^ and ν(*Si–H_3_) 2133 cm^–1^. Furthermore, the region between 600–700 cm^–1^ marked as δ(*Si–H_*x*_) is characteristic of *Si–H bending and/or *Si–H_2_ wagging modes.^35,40^ However, the assignment of the doublet marked as δ(*Si–H_3/2_) at 905 and 857 cm^–1^ is less clear. Based on literature data, it can represent the degenerate and symmetric deformation modes of silicon trihydrides *Si–H_3_, respectively, even though also silicon dihydrides can be present in this region.^34^ Unlike the two other features, this doublet appears only in plasma-synthesized Si nanoparticles,^3,34,36,41^ whereas in SiQDs prepared by electrochemical etching or by thermal annealing of Si-rich SiO_2_, only a single peak at ≈900 cm^–1^ appears.^35,39,40,42^ Thus, we believe that the spectral shape of the 900 cm^–1^ region is, for reasons currently unbeknownst, linked to the applied synthesis method rather than to the structural properties of the sample. One possibility is that one of the peaks in the doublet is not directly connected to the surface of Si nanoparticles, but rather represents the interconnecting material.

Interestingly, the structural change from fully amorphous to structurally ordered material evidenced by Raman measurements in Figure 1b is also reflected on the surface of the nanoparticles, as confirmed by FTIR measurements. The FTIR stretching-mode region at 2000–2200 cm^–1^ and the deformation modes at 600–700 cm^–1^ (Figure 1c) exhibit a systematic change from broader smooth peaks in the amorphous nanopartices to sharper lines and a more structured shape in the structurally ordered SiQD sample. Narrowing of peaks in a crystalline SiQD is to be expected, since the more ordered character of the QD core also causes higher ordering of the surface of the QD, resulting in better-defined vibrations of surface hydrides. The narrowing effect in the SiQD sample is evident especially in the deformation-mode δ(*Si–H) spectral region, where a clearly resolved doublet appears at 630 and 661 cm^–1^, together with a hint of a side shoulder at 695 cm^–1^. Here, the 630/661 cm^–1^ doublet is likely to originate in *Si–H bending deformations and the side shoulder at 695 cm^–1^ is due to *Si–H_2_ wagging vibrations. A very similar spectral shape of the corresponding FTIR peak is typically observed also in fully crystalline SiQDs in porous-silicon layers.^35,40^ Unlike these spectral regions, the 905/857 cm^–1^ doublet stays resolved in all the samples regardless of their degree of structural order. This absence of spectral change is likely connected to the unclear assignment of this spectral structure as discussed above. Therefore, this structure will not be further discussed in the text. Notwithstanding, our experiments clearly demonstrate that the structural ordering of SiQDs is strongly reflected also in their surface-bond vibrations.

Next, we studied the ignitions conditions for the amorphous Si nanoparticles and SiQDs from Figure 1b,c. The samples were ignited as deposited on the glass slide. The glass slides were either placed on a hot plate and slowly heated up, or they were irradiated with a 405 nm (50 mW) laser pointer with increasing the intensity of the laser pointer and the conditions required for ignition were recorded. The results are presented in Figure 1d. An important observation here is that the ignition temperature is relatively low, in the range of 150–200 °C, which is even below the dissociation temperatures of higher hydrides on silicon, as shown later in Figure 4b. Moreover, this is a much lower temperature than that reported when amorphous Si nanoparticles were used as an additive to lower ignition temperature in an Si/KClO_4_ nanocomposite.^3^

As the next step, we wanted to test if and how much the ignition properties of Si nanoparticles can be influenced by the surface. Thus, we synthesized “amorphous 2” Si nanoparticles close to the amorphous/structurally ordered threshold, but using a varying H_2_ dilution in the synthesis gas. Diluting the synthesis gas with H_2_ induces an etching-like process in the plasma and thus can change the composition of surface hydrides.^34^ The FTIR spectra of these Si nanoparticles in Figure 1e show very little difference with increasing H_2_ dilution, demonstrating that due to the amorphous nature of these nanoparticles, potential changes in the overall composition of the surface hydrides are small. However, the subsequent study of the ignition conditions presented in Figure 1f reveals a systematic trend of decreasing ignition temperature and light intensity with increasing H_2_ dilution. This direction of the trend is intuitively expected, as nanoparticles with more disturbed surfaces due to the additional hydrogen in the synthesis process are likely to be less stable. However, quantitatively speaking, the corresponding changes are modest, about 5% at most, corroborating the nearly identical FTIR spectra.

Thus, even though the properties of the surface have a clear potential to be used to tune the thermal properties of nanoparticles, amorphous nanoparticles do not seem to be the ideal candidate. Therefore, a more thorough study of the surface composition and thermal properties was conducted using structurally ordered QDs.

### Structural and Surface Characterization of SiQDs

Therefore, we synthesized a set of highly structurally ordered Si nanoparticles with varying H_2_ flow in the synthesis gas and characterized both their structure and surface chemistry. Then, to address the suitability of Si nanoparticles for energy-storage applications, we measured the energy density released by these nanoparticles during oxidation.

However, one possible interpretation of the low-temperature ignition mechanism in the sample of Si nanoparticles we reported in Figure 1 is the release of energy caused by the contact of hydrogen trapped in the microvoids within the pile of Si nanoparticles with atmospheric oxygen. Thus, the experiments presented in this section were performed on “fresh” and “aged” samples. In “fresh” SiQDs, the measurements were conducted relatively shortly (24 h at maximum) after the synthesis with minimized exposure to atmospheric oxygen. Samples referred to as “aged” were kept approximately 14 days in a nitrogen-filled glovebox to significantly decrease the potential hydrogen trapped in microvoids. Thus, the differentiation between the “fresh” and “aged” samples will reveal the influence of both potentially trapped hydrogen and storage time on the studied SiQDs.

**Figure 2:**
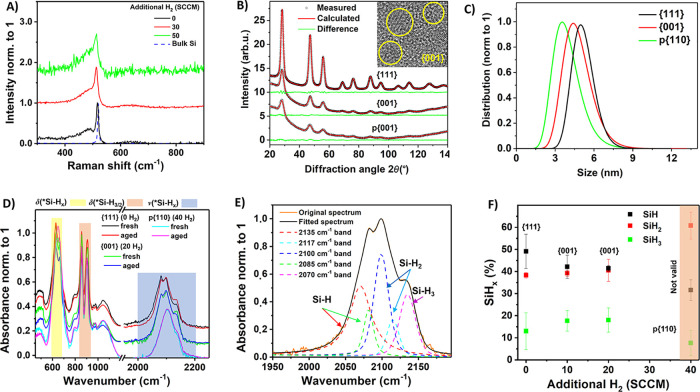
(a) Raman spectra of SiQDs synthesized under varying hydrogen dilution. (b) Measured (black circles), calculated (red line), and difference (green line) X-ray diffraction patterns of the structurally ordered SiQDs with varying hydrogen dilution (please note that a smaller amount of {001} and p{110} sample was used due to technical reasons). Representative examples of the nanoparticles characterized by HRTEM are shown in the inset. (c) Particle size distributions of the sample from panel (b) determined from SAXS measurements, the corresponding fits are shown in Figure S5. (d) FTIR spectra of the samples from panel (b). Samples marked as “fresh” were characterized shortly after the synthesis, samples marked as “aged” were characterized after about a 14-day storage in a nitrogen-filled glovebox. Full FTIR spectra are shown in Figure S7. (e) Example of the deconvolution of the spectral structure ν(*Si–H_*x*_) into the individual components for the 20-sccm SiQD sample. (f) Ratio of silicon mono-, di- and trihydrides on the surface of SiQDs from panel (b) determined using the deconvolution shown in panel (e). “Fresh” and “aged” samples are treated simultaneously since no difference was detected in their FTIR spectra. The error bars correspond to standard deviations. The FTIR spectra of the p{110} sample are no longer well-resolved due to a lower degree of structural order on the surface, which invalidates the deconvolution analysis in that sample.

In analogy to the experiments from Figure 1, the character of the surface of structurally ordered SiQDs is affected by hydrogen flow in the synthesis gas. The influence of hydrogen flow was first probed using Raman spectra of SiQDs, as presented in Figure 2a. Clearly, if hydrogen flow is kept under 50 sccm, the synthesized nanoparticles are highly structurally ordered, as evidenced by the relatively narrow Raman line at ≈514–519 cm^–1^. Therefore, in order to focus on structurally ordered SiQDs, hydrogen flow during the synthesis of SiQDs was varied in the 0–40-sccm range.

**Figure 3:**
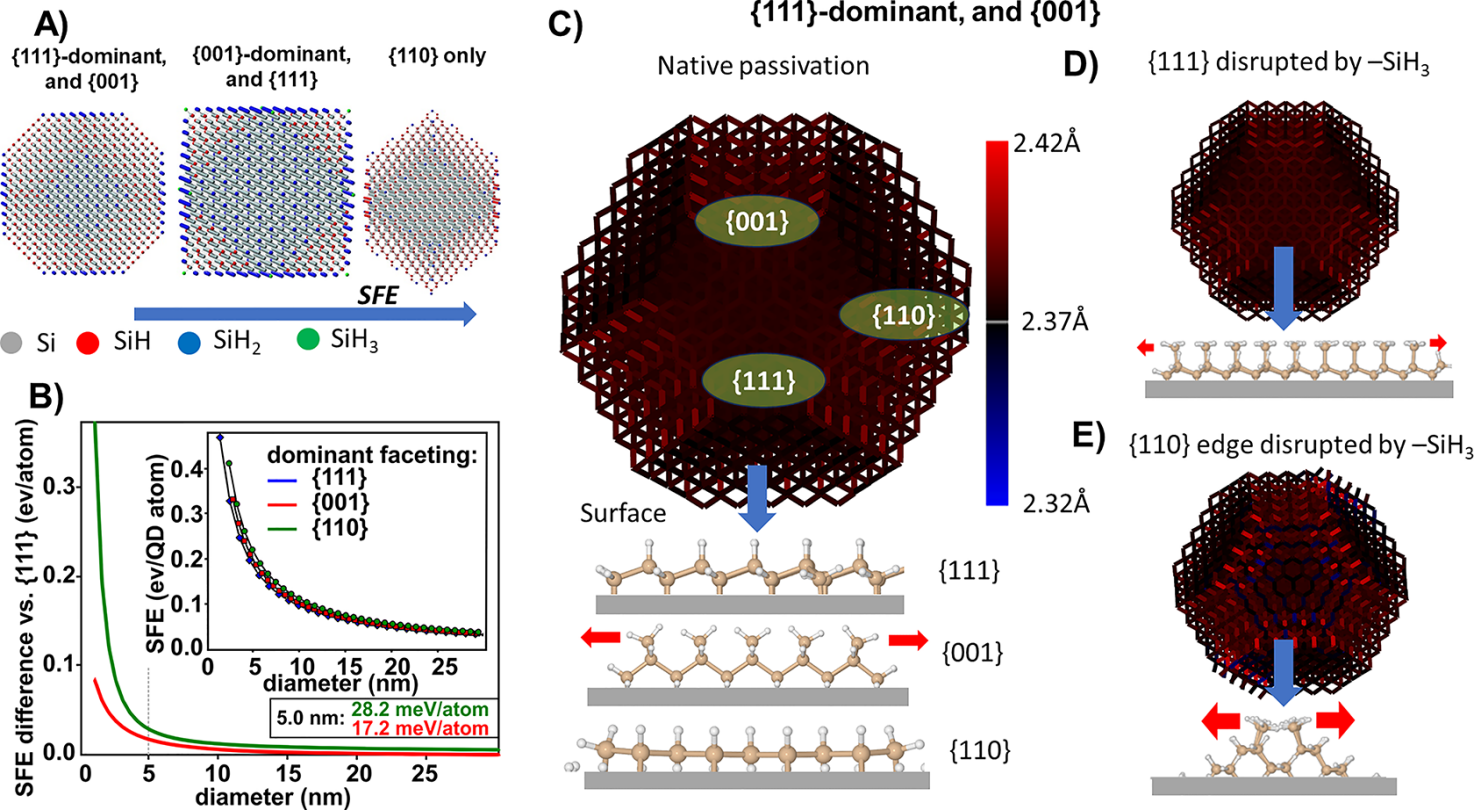
(a) Idealized geometric models of SiQDs cut off from a perfect crystal so that certain types of crystalline facets on the surface are exposed. The color coding highlights the different ratios of silicon hydrides in such structures. Adapted after Ref (6) (CC BY license). (b) Difference in SFE Δ*SFE* relative to the {111}-dominant faceted QD type with the lowest SFE values. The inset shows absolute values from which Δ*SFE* was derived, see text for details. (c) Example of a geometry of a 5 nm natively H-passivated SiQD with {111} dominant faceting relaxed by the classical force field. The color of the bonds indicates the difference of bond length from the bulk lattice constant, illustrating passivation-induced strain. The bottom of the panel shows native bonding of hydride surface groups on different facets. (d) Geometry from panel (c) was disrupted by the incorporation of *SiH_3_ groups, simulating the extreme situation, where exactly half a layer of Si atoms was etched away by hydrogen plasma. Only minor strain is induced. The bottom of the panel shows hydride bonding on the disrupted {111} facet. (e) Geometry from panel (c), where Si–Si bonds at the {110}-like edge are broken and replaced by hydrogen to simulate the plasma etching, leading to *SiH_3_ groups. Significant strain is induced. The bottom of the panel shows hydride bonding on the disrupted {110}-like edge.

**Figure 4:**
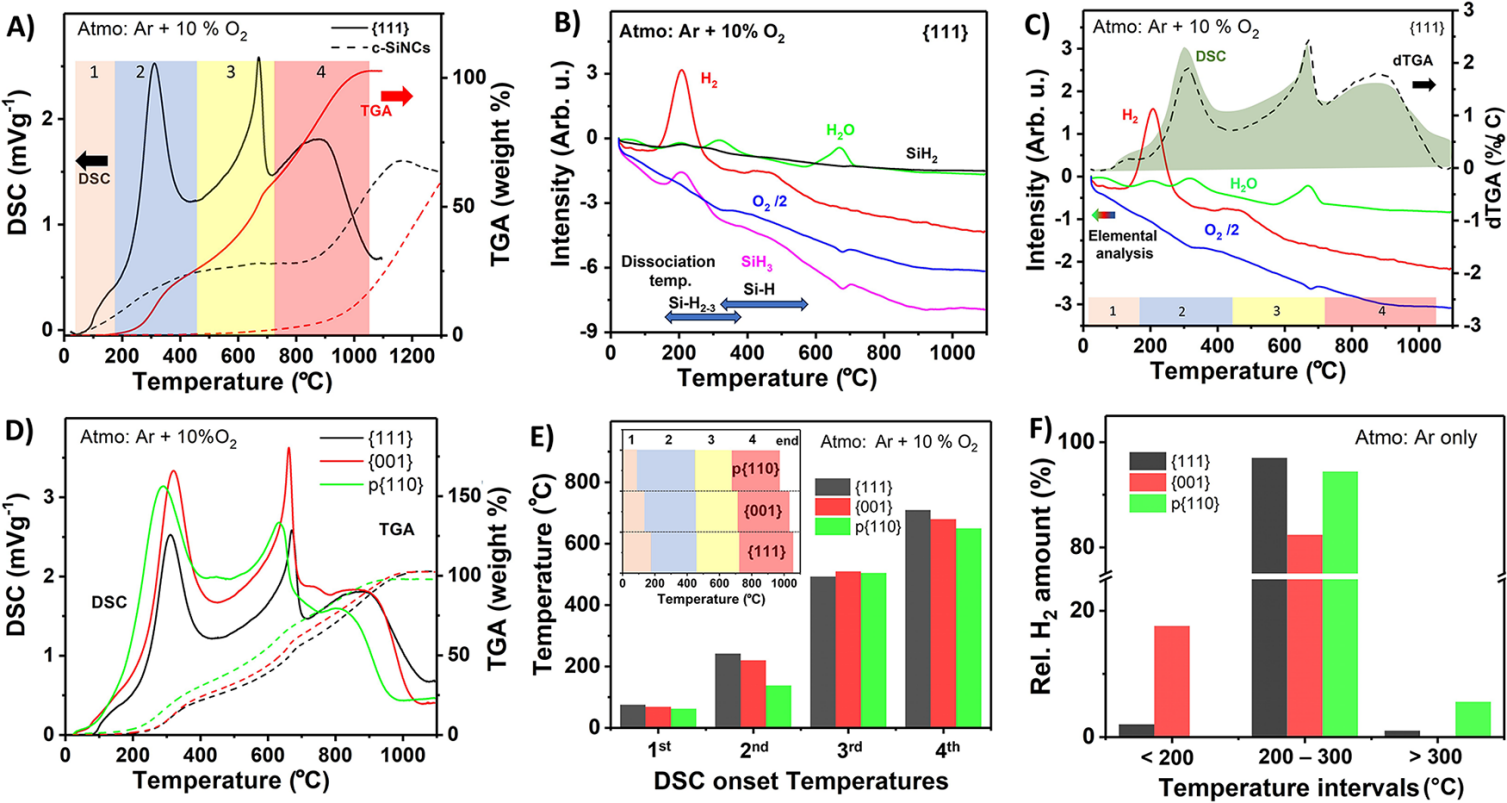
Characterization of the thermal properties of SiQDs by simultaneous measurements of differential scanning calorimetry (DSC), thermogravimetric analysis (TGA), and mass spectrometry (MS). (a) DSC and TGA measurements of SiQD-{111} (solid curves) and commercial Si nanocrystals (170 nm, the dashed curves). The studied temperature range is divided into four zones based on the prevailing effects responsible for the release of energy. (b) Elemental analysis of selected species released/consumed during the annealing of SiQD-{111}. Ranges of reported dissociation temperatures for *Si–H_*x*_ species are also included.^3,26,38,55,56^ (c) Combination of the DSC and MS curves from panels (a) and (b) plotted in one graph to facilitate the comparison. These curves are complemented with a derivative of the TGA curve (dTGA) from panel (a). (d) Comparison of the DSC and TGA curves of the three studied SiQD samples. (e) DSC-based onset temperatures marking the onset of energy release in the four zones identified in panel (a) for the three studied SiQD samples. The inset directly compares the TGA-derived temperature ranges marking different oxidation processes, corresponding to the individual zones in the studied samples. (f) Origin of released hydrogen for the three studied samples. The analyses presented in all panels were performed in an oxidizing (10% O_2_/Ar) atmosphere, with the exception of panel (f), which reports released hydrogen under an inert atmosphere (pure Ar), where the presence of oxygen cannot interfere with its detection.

X-ray diffraction (XRD) measurements were used to obtain more detailed structural and microstructural information about SiQDs synthesized under varying hydrogen flow. Measured XRD patterns fitted using the whole powder pattern fitting procedure, specifically the Rietveld method using the MStruct software,^43^ are shown in Figure 2b. XRD confirmed that the investigated samples consist of a single crystalline phase, namely cubic Si (space group *Fd*3̅*m*). The significant broadening of the diffraction profiles in comparison to bulk Si clearly confirms nanocrystalline nature of the investigated samples. The refined lattice parameters presented in Table 2 are only slightly smaller (0.2–0.5%) than the tabulated data for coarse-grained, defect-free standard Si powder. The size of coherently diffracting domains was modeled using a log-normal distribution and mean sizes are listed in Table 2.

**Table 2:** List of Mean Diameters of the Investigated Samples Determined Using Different Methods, and  Is Used as the Error to Characterize the Width of the Distribution^a^

SiQD sample	mean diameter (nm)			*a* (Å)
	SAXS	HRTEM	XRD	XRD
{111}	5.5 ± 1.0	6.2 ± 0.5^b^	4.0 ± 0.2	5.4258 ± 0.0014
{001}	4.6 ± 1.2	5.5 ± 0.7	3.2 ± 0.3	5.4269 ± 0.0007
p{110}	4.0 ± 1.5	4.0 ± 0.7	2.4 ± 0.3	5.4282 ± 0.0002

a The last column lists lattice constants *a* derived from XRD characterizations (NIST Si standard reference material: *a* = 5.43102 Å).

b The HRTEM-derived distribution of the {111} sample indicates the presence of smaller sizes of (4.8 ± 0.4) nm making up approximately 15% of QDs.

The distribution of sizes was determined using small-angle X-ray scattering (SAXS) by fitting using the model of spherical particles with the sticky hard sphere structure factor^44,45^ assuming the log-normal distribution. Refined normalized log-normal distributions of particle diameters are shown in Figure 2c. Additionally, the sizes were also characterized using high-resolution transmission electron microscopy (HRTEM), as shown in Figure S6. Mean sizes derived using these two methods are listed in Table 2. Clearly, the SAXS- and HRTEM-derived sizes are in good agreement. There is a significant overlap between the distributions of sizes of the individual samples, which can be quantified using the Bhattacharyya coefficient of the correponding normal distributions as 0.75 and 0.88 for the first and second, and second and third sample, respectively. Thus, even though the mean sizes decrease from about 5.5 to 4 nm with increasing hydrogen flow, this difference is not large considering the significant overlap of the distributions.

Mean sizes were also determined from XRD measurements, yielding smaller values than the SAXS- and HRTEM-based distribution. This difference is a consequence of the applied method, since XRD detects only the signal from the volume diffracting with a high degree of coherence, which is evident from the high certainty of the derived lattice constants in Table 2. Thus, in contrast to XRD, HRTEM and SAXS also include the reconstructed surface. This effect has been observed in the past and was attributed to an amorphous shell.^46^ However, in our case, the surface of the investigated samples has a higher degree of structural order when compared to a purely amorphous layer, as will be shown below. Notably, in addition to the good agreement between the size distributions obtained by different methods, our measurements confirm the absence of potential larger nanoparticles, which could influence the results of further experiments.^47^ Both the SAXS and XRD techniques probe macroscopic amounts of SiQDs, ensuring that the derived distributions represent the whole sample. Combining the macroscopic characterization with probing of the whole core of QDs, SAXS is the method of choice for the accurate determination of size distributions, while HRTEM guarantees the accuracy of the absolute size measurements obtained.^48^

The Fourier-transform infrared spectroscopy (FTIR) characterization of the surface of SiQDs synthesized under varying hydrogen flow is shown in Figure 2d. First, there is only the negligible difference between the “fresh” and “aged” varieties of the samples, including a low level of oxidation evidenced by the weak ≈1045 cm^–1^ Si–O–Si peak. Thus, we can conclude that medium-term storage time in nitrogen atmosphere at room temperature does not affect the surface of the synthesized SiQDs to a notable degree. Furthermore, the SiQDs synthesized in the 0–30-sccm H_2_ flow keep a very similar well-resolved spectral structure in both the δ(*Si–H_*x*_) bending-deformation spectral region at 600–700 cm^–1^ and at the ν(*Si–H_*x*_) stretching-mode region at 2000–2200 cm^–1^, except for a somewhat different ratio of the mono-, di- and trihydrides.^26,37^ Following the well-established methodology presented in Ref (26) we deconvoluted the ν(*Si–H_*x*_) stretching-mode region into the individual components, as outlined in Figure 2e.

The results of the surface-hydride analysis are presented in Figure 2f. This analysis confirms that Si trihydrides are the least common ones and that there is a roughly 1:1 ratio of dihydrides and monohydrides on the surface of SiQDs. However, a clear step-like change is detected with the addition of hydrogen flow to the synthesis, when the relative ratio of surface monohydrides drops in the 10-sccm sample but stays the same for the 20-sccm sample. However, in the 40-sccm sample, a clear change of the shape of the FTIR spectrum occurs: the typical structure at the bending-deformation spectral region at 600–700 cm^–1^ disappears and the peak slightly shifts, see Figure 2d. Moreover, the lines in the 2000–2200 cm^–1^ stretching-mode range broaden and merge. Whereas the typical FWHM of the fitted lines is 25 cm^–1^ in the 0–30-sccm samples, apart from the first line wider probably as a result of fitting of the minuscule tail at 2000 cm^–1^, fitted peaks reach the FWHM of 40 cm^–1^ and are much less proportionate in the 40-sccm sample. Overall, the broader FTIR peaks and change in spectral shape prohibit the automatic application of the same deconvolution approach, as the surface of this SiQDs clearly reverts to a less-ordered state despite the structurally ordered core confirmed by Raman spectroscopy. Even though the changes observed in the 2000 cm^–1^ spectral region used for the surface-hydride analysis are small, repeatability of the obtained results ensures that our approach is valid.

To summarize, the structural characterization presented in Figure 2a–c proves that the SiQD samples studied here are, given the state-of-the-art synthesis protocols in this material, as similar as possible in their internal structure, whereas their surface properties can be tuned (Figure 2d–f) by changing hydrogen flow in the plasma during synthesis. The three different types of QDs support a different composition of surface hydrides and also likely a structurally less ordered state in the sample synthesized with the highest hydrogen flow. Also, no detectable differences were found between the “fresh” and “aged” varities of the sample, implying that no trapped hydrogen is present in the porous samples and that storage time in a nitrogen-filled glovebox before the measurement does not influence the surface properties.

### Dominant Faceting

The quantitative and qualitative interpretation of FTIR spectra can be combined using models of SiQDs. In general, models of SiQDs can be computationally constructed with the structures being optimized to attain the lowest total energy state simulating surface reconstructions, but such calculations are extremely computationally costly and have so far been performed only for smaller QDs.^49^ However, as a first approximation, we can use a simple purely geometrical models of ideally crystalline SiQDs constructed by being cut off from a perfect crystal and exposing certain facets,^6^ see Figure 3a. Clearly, a certain type of facet prevails on the surface of these QD models, which we refer to as dominant faceting. The SFE area densities of the relevant facet orientations are  J/m^2^,  J/m^2^, and  J/m^2^.^50^ Using the analytic framework of Ref (6) we can derive the surface area per facet orientation for all three SiQD types as a function of their size, see Section S1 in Supporting Information. The multiplication of the above SFE density values with such specifically oriented surface areas yields the total SFE per SiQD. The nominal unit for such SFEs at hand would be femto-Joule (fJ) which is somewhat difficult to relate to general forces in the nanoscale range such as van der Waals or Casimir–Polder forces. Therefore, we relate the SFE to the number of Si atoms forming the respective QD, and convert the energy units from fJ to electronvolt per atom. This way, we obtain an SFE value per QD atom which can readily be related to general nanoscale forces.

For the three SiQD topologies from Figure 3a, we show the SFE values in the inset of Figure 3b. For comparing different QD geometries, we need the difference in SFE (ΔSFE) *relative* to a baseline given by the QD type with the lowest SFE values, see Section S1 for details. This QD type is presented by the quatrodecahedral SiQD with {111}-dominated faceting, see Figure 3b. For QD sizes of around 5 nm as featured in our work, we obtain a total SFE per QD of SFE_q.111_ ≈ 0.18 eV/QD atom for the {111}-dominated geometry, of SFE_q.001_ ≈ 0.20 eV/QD atom for the {001}-dominated geometry, and of SFE_q.110_ ≈ 0.22 eV/QD atom for the {110}-dominated geometry. Due to the selectivity of chemical reactions, each type of facet supports a certain type of hydride configuration as becomes apparent from geometrical boundary conditions, see Figure 3c.

First, these SiQD models show a very low proportion of trihydrides, which appear only at the “apexes”. The low proportion of trihydrides is corroborated by our experiments, even though the measured proportions are higher than what would be expected from the idealized geometry. This finding can be interpreted to the end that the increased amount of detected *SiH_3_ accounts for a non-negligible structural disturbance on SiQD surfaces, forming small terraces with numerous edges and atomic steps.^26^ Second, as demonstrated by the analytic QD description in Ref (6) a model QD with dominant {111} faceting is terminated dominantly with monohydrides, but dihydrides are still non-negligible. The ideal dominantly {001} faceted QD contains roughly a balanced proportion of mono- and dihydrides, while exclusive {110} faceting leads to a very high proportion of monohydrides on the surface. Importantly, the very same pattern of the evolution of the composition of hydrides occurs in our synthesized SiQDs. In the sample synthesized without any hydrogen flow, monohydrides are dominant according to Figure 2f and the 2080 cm^–1^ FTIR line corresponding to hydrogen on Si{111} in bulk is the strongest. In the samples synthesized with up to 30 sccm hydrogen flow, mono- and dihydrides are roughly balanced, similarly to the ideal geometrical model with dominant {001} faceting. The single broader band at ≈2100 cm^–1^ in the 40-sccm sample, where the mathematical deconvolution is no longer valid, most likely corresponds to the vibrational bands of monohydrides on several orientations of crystalline Si surfaces, possibly with the dominance of Si{110}.^51,52^ The interpretation of an increased proportion of monohydrides incurred by H_2_ etching in plasma in the 40-sccm sample is supported by the disappearance of its *Si–H_2_ shoulder line at 695 cm^–1^ at this sample.

Thus, even though the variability in the composition of surface hydrides had been reported in the past,^26,51^ we provide a physical interpretation of this phenomenon. The additional H_2_ flow in plasma, causing “etching” of the surface of SiQDs and/or influencing the transfer of kinetic energy from Ar ions during the collisions with the forming particles, leads to different dominant faceting of the synthesized QDs, evolving as {111} → {001} → {110} with increasing H_2_ flow in the synthesis gas. Our samples will be labeled using this notation, see Table 1, apart from the 40-sccm sample, which will be referred to as p{110}, where p stands for partial faceting due to the likely more disordered nature of its surface.

This classification based on dominant faceting does not imply that each of the synthesized SiQDs in the particular sample has the shape shown in Figure 3a. Since the synthesis is a highly nonequilibrium process, the forming nanoparticles typically do not reach ideal faceted crystal structures. During the formation process, apexes and edges are the least stable parts, most accessible to potential cleavage. Therefore, the surface is likely made up by a larger number of facets, forming smaller terraces.^26^ Such a multifaceted nanoparticle, albeit assuming embedding in an SiO_2_ matrix in their case, was constructed theoretically by Hadjisavvas et al.^53^ Their calculation produced a 5 nm SiQD with 42 facets of three types. In our experimental study, we propose an analogous situation: we argue that a realistic QD, unlike the idealized models from Figure 3a, typically has a larger number of facets, with edges and apexes possibly eroded.^24^ Such a complicated polyhedral shape is basically indistinguishable from a spherical one using classical HRTEM imaging, let alone for a macroscopic number of QDs. However, a certain type of the exposed surface is dominant. We propose that, when experimentally probing a macroscopic set of QDs, the preference for a certain type of surface, or dominant faceting, is the key property *regardless of the spatial distribution of these facets on the surface of the QD*. Thus, a set of SiQDs with one type of dominant faceting will exhibit an average characteristics in analogy to the corresponding bulk material, e.g. in its thermal properties.

During the formation of a nanoparticle, the details of the quasi-spherical polyhedral shape are a result of the local minimization of strain energy originating in bond-length and bond-angle distortions. A thorough analysis of the synthesis process and atomistic details of the surface of realistic particles are beyond the scope of the current article. Nevertheless, it is fairly certain that the limited reaction time, energy, and varying volume flux density of the SiQD precursor SiH_4_ in the nonthermal plasma results in different SiQD geometries, of which many are “frozen” in a premature stage by the limited time-energy integral during their formation. To gain some insight into the relation between crystalline facets, surface passivation and the formation of the nanoparticles in hydrogen-enriched plasma, we calculated local strain of Si–Si bonds induced by the presence of different hydrides for three limiting model situations using the classical force field simulations based on FireCore.^54^ First, a {111}-dominantly faceted SiQD with the diameter comparable to the QDs we synthesize was constructed (see Figure 3c). In addition to {111} facets, this model also has {001} facets to mimic the observed spherical shape. The edges between the {111} and {001} facets are structurally equivalent to {110} facets. Already in this natively passivated model, the less stable {001} facets are slightly tensile-strained by the steric repulsion of the natively passivating *SiH_2_ groups, whereas the {111} facets are completely bulk-like. To simulate hydrogen “etching”, this geometrical model was disrupted by removing the topmost Si atoms and artificially repassivating the bonds with *SiH_3_ groups. When such disruption was applied to the {111} facet, see Figure 3d, the passivating *SiH_3_ groups sterically fit rather well and produce only minor strain, comparable to or even smaller than that in natively *SiH_2_-passivated {001} facets. We do not expect the presence of *SiH_3_ groups at {001} facets as the removal of an Si layer at this facet recovers the *SiH_2_-pasivated {001} surface (just rotated by 90°). However, the disruption of the {110}-like edge produces sterically hindered *SiH_3_, see Figure 3e, which face each other due to the geometry of the underlying crystal lattice, causing significant tensile strain to nearby Si–Si bonds. In realistic nanoparticles, this local strain would make these bonds more reactive and prone to further etching and can even lead to reorganization of the surface. Thus, we put forward a hypothesis that the local strain, especially at the {110} edges, leads to the lower thermal stability and increased reactivity of hydrogen-“etched” SiQDs as well as to the observed nonideal quasi-spherical shape.

The different dominant faceting is associated with increasing surface energy *E*_{xyz}_, with *E*_{111}_ < *E*_{001}_ < *E*_{110}_(6,50) and with increased local strain on the facets. Therefore, we predict that the presence of H_2_ in the synthesis gas leads to SiQDs structures with higher SFE and thus lower thermal stability. The first piece of evidence supporting the lower thermal stability of higher-SFE samples is the measurement of ignition temperature of the SiQD samples shown in Figure S8. These measurements clearly indicate that lower-SFE samples are systematically ignited at lower temperatures. The lower thermal stability is also supported by the analytic SFE calculations above which clearly show a maximum SFE for the {110} facets. Therefore, SiQDs with an increased fraction of {110} facets become thermally less stable and are prime candidates for a pyrophoric reaction in air at the minimum possible temperature.

In the theoretical study by Hadjisavvas et al.,^53^ the {001} interface was found to be the most stable one because they consider a system of SiQDs embedded in surface-bonded oxide. Here, we focus on free-standing hydrogen-terminated SiQDs. As hydrogen termination is known to influence the electronic properties of SiQDs to only a very small extent,^49^ the stability trend we propose in our system reflects the purely structural SFE of silicon surfaces.^50^ Thus, the term of dominant faceting we introduce here, using the case study of hydrogen-terminated SiQDs, can be generalized to other systems. In ligand-terminated QDs, a prevalent type of crystallographic surface in a highly polyhedral, quasi-spherical QD can be linked to the composition of the surface ligands as in our case. In more complicated systems, the difference in interfacial energy corresponding to individual facets will be minimized, but the quasi-spherical shape with dominant faceting can still prevail.

### Energy Density

For assessing the energy density of our synthesized SiQDs, we carried out calorimetric measurements in pure oxygen, see Figure S9. The calorimetric measurements were performed separately for the “fresh” and “aged” varieties of the samples, but no statistically significant differences were observed. Thus, the “fresh” and “aged” varieties of samples were grouped together. (One exception was the p{110}-fresh SiQD sample, which burnt when introduced to the calorimetric chamber and therefore could not be characterized.) The measured values of released energy (32.0–32.6 kJ/g) are equal to the theoretical limit of the gravimetric energy density in silicon, confirming that all the silicon present in the sample is indeed surface-oxidized, as has already been suggested by the clear white color of the Si nanoparticles after combustion in Figure 1a. As a comparison, we also performed calorimetric measurements of commercial Si nanocrystals with the diameter of about 170 nm, which, in contrast to SiQDs, burnt only incompletely and their released energy was lower by about 15%, see Figure S9. Thus, reaching the theoretical limit of released energy in SiQDs was made possible by the nanometer-sized nature of the sample with its high surface-to-volume ratio.^6^

The calorimetric measurements of oxidized, as opposed to hydrogen-terminated, SiQD-{111} sample showed a modest decrease in released energy to 30.5 kJ/g. The decrease is easily understandable because in an oxidized SiQD sample, the oxidized surface bonds cannot release any energy. Using a simple estimate based on a geometrical model,^6^ about  of bonds in a 5 nm SiQD are interfacial and the energy contained in the in the “surface” Si–H bonds makes up about 3–5% of the energy originating in the “volume” Si–Si bonds, depending on dominant faceting. In accordance with this estimate, the energy density of the H-terminated samples is by about five percent larger than that of the oxidized sample. This estimate also indicates that the relative differences of the gravimetric density based on varying dominant faceting and different content of hydrogen are very small, in the range of 1–2%, again in accordance with Figure S9. Thus, in our set of samples, nearly the same amount of energy is released during oxidation, regardless of dominant faceting and the composition of surface hydrides. However, as a result of the highest density of surface bonds,^6^ the relatively highest amount of Si–H-originating energy (5%) should be present in the {001}-dominated sample. Again, Figure S9 suggests the very same tendency, even though the difference is close to the experimental error. Also, the {001} facets chemisorb the highest amount of hydrogen, whereby it is also plausible that such H atoms initiate the oxidation process in SiQDs.

### Thermal Properties of SiQDs

In order to understand the phenomena responsible for the flammability of SiQDs presented in Figure 1 and to verify if the dominant faceting introduced in Figure 2f and SFE influence the thermal properties of SiQDs, we performed *simultaneous* differential scanning calorimetry (DSC) measurements, thermogravimetric analysis (TGA) and mass spectrometry (MS). As no detectable differences between the “fresh” and “aged” varieties of the samples were found, the storage time before measurement was disregarded as a potential parameter and the “fresh” and “aged” varieties were no longer distinguished. These measurements were conducted in the temperature range from 25 to 1100 °C (unless specified otherwise) in both, 10% O_2_/Ar atmosphere, referred to as “oxidizing”, and pure Ar atmosphere, referred to as “inert”. As samples, we used the three types of SiQDs and the commercial SiNCs listed in Table 1.

Prior to the discussion of the SiQD samples, it is important to discuss the effects present in a typical commercial nanostructured Si (c-SiNCs, diameter 170 nm), which does not exhibit low-temperature ignition and is surface-oxidized. Its behavior during annealing under oxidizing atmosphere is shown in Figure 4a by the dashed curves. The slowly increasing DSC curve with two visible broad bands with onset temperatures of 80 and 830 °C confirms a slow gradual release of energy during annealing. Moreover, the increase of the TGA curve implies that the c-SiNCs sample gains weight. Initially, the increase of mass is only by about 6 (w/w)% in the temperature range 300–800 °C, and the subsequent significant rise continues to 1350 °C, reaching 64 (w/w)%. This sample is not fully oxidized after the annealing. MS measurements did not detect any signal related to any monitored compounds or fragments except for the ongoing consumption of oxygen (see Figure S10). Thus, as the sample underwent the initial surface oxidation, the only two effects observed in c-SiNCs during annealing are connected with (i) the slightly exothermic structural rearrangements of surface oxide at temperatures <800 °C and (ii) the diffusive oxidation of the Si core at high temperatures (800–1350 °C). This interpretation is further validated by the thermal characterization of surface-etched partially hydrogen-terminated commercial Si nanocrystals (H-c-SiNCs). The corresponding DSC and TGA curves are shown in Figure S11 and the MS curves are presented in Figure S10. During annealing, the DSC and TGA curves of H-c-SiNCs followed a similar trend as the one observed in oxidized c-SiNCs under oxidizing atmosphere with virtually no effects in the whole 25–1000 °C temperature range under inert atmosphere. One subtle difference is a hint of an exothermic effect in the DSC curve starting at around 250–300 °C, see Figure S11a, which is likely connected with the surface dehydrogenation and oxidation of this sample. Moreover, the diffusive oxidation effect clearly starts and finishes at lower temperature for the partially oxidized nanocrystals (H-c-SiNCs), which is due the thicker oxide shell in c-SiNCs, representing a higher barrier for the oxidation process. These results corroborate the well-known stability of silicon structures, in which the bulk of the material is susceptible only to diffusive oxidation at high temperatures.^15,16^ Clearly, this stability persists even at relatively small sizes of the nanostructures of about 170 nm.

In contrast to simple nanostructured Si, SiQDs show a much wider variety of processes during the annealing process. As illustrated in Figure 4a for the SiQD-{111} sample, the studied temperature range can be divided into four different zones. The onset temperatures of the weight-gaining processes (oxidation) in the TGA curve mark the end points of the intervals: Zone 1 ranges from 25 to approximately 180 °C with no increase in weight of the sample, followed by Zone 2 from 180 to 450 °C with 25 (w/w)%, then followed by Zone 3 between 450 and 720 °C with a 35 (w/w)% increase and, finally, Zone 4 from 720 to 1050 °C with a 44 (w/w)% increase. After heating, the SiQD sample is fully oxidized, as corroborated by the change in its ambient-lightning color from beige-orange to white, see Figure S12. The total relative weight increase during the heating process reaches 104 (w/w)%, which is slightly lower than the theoretical value of 114 (w/w)% in an ideal sample made up solely by silicon atoms undergoing complete oxidation. As a reference, TGA measurements of the SiQD-{111} sample under inert atmosphere showed only minor changes, see Figure S11d, namely a small decrease of about 1 (w/w)% in the interval from 235 to 500 °C, which can be explained by the release of hydrogen, and a slow continuous relative weight rise of about 2 (w/w)% up to 1100 °C (Figure S13a), which likely results from weak oxidation caused by either adsorbed water or trapped oxygen, or tentatively impurities in the carrier Ar gas (Figure S13c).

**Figure 5:**
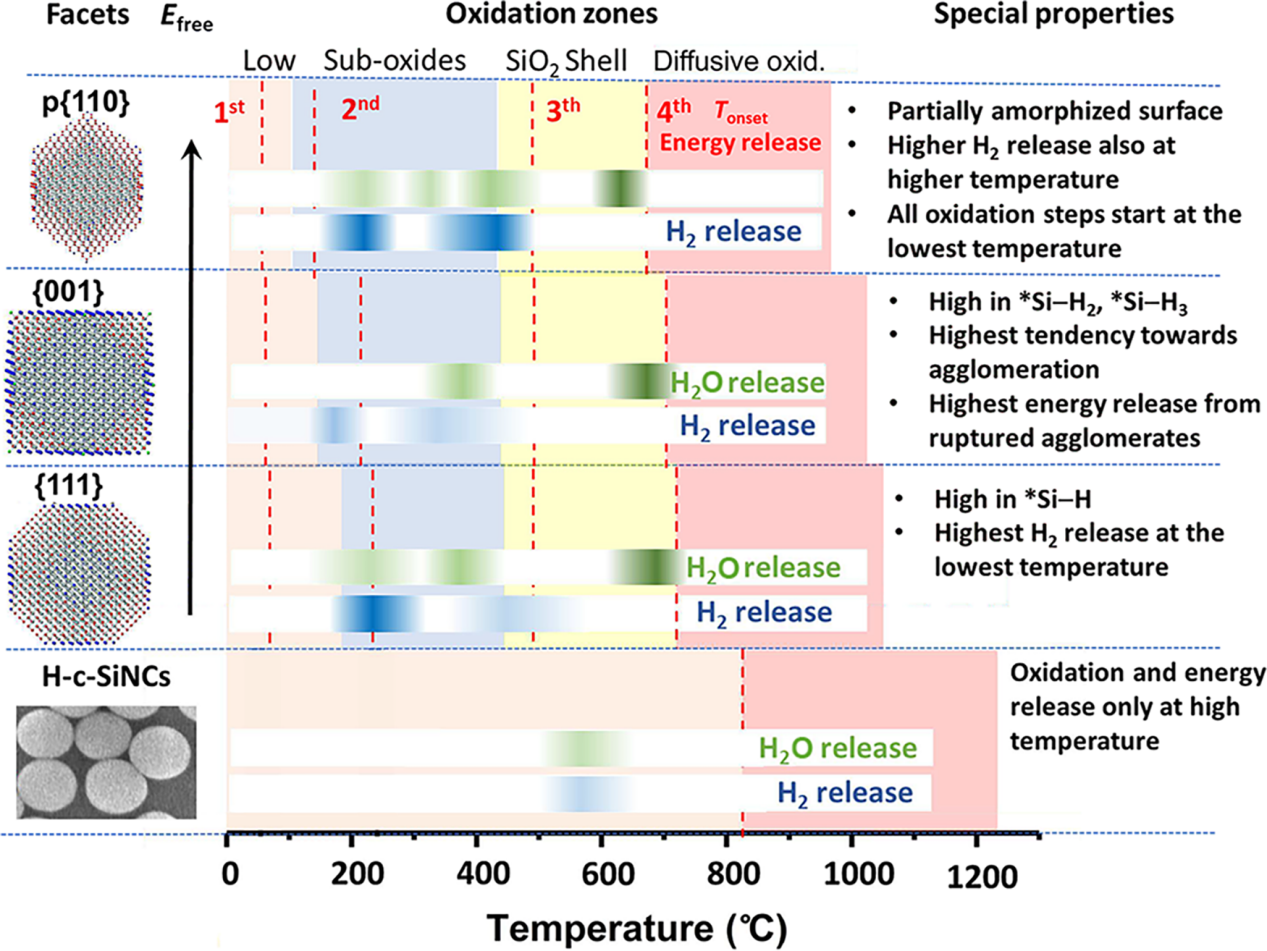
Schematic summary of the observed trends in SiQDs compared to commercial partially hydrogen terminated c-SiNCs, serving as a guide for tuning energy storage properties and its release. The different TGA-based oxidation zones are shown by different colors, the energy-release DSC onset temperatures are shown as the red dashed lines. Temperature ranges in which hydrogen is released are indicated by the dark blue stripes. Detection of water, most of which originate from released hydrogen reacting with oxygen before detection, is marked by the green stripes. The detection of solely water in the 600–700 °C range in the SiQD samples comes from hydrogen released from ruptured agglomerates. The main properties of the individual samples are listed. Please note that the H-c-SiNC sample also very likely undergoes a small degree of oxidation around 600 °C, where released hydrogen is detected. However, due to a much smaller relative surface-to-volume ratio in these large nanoparticles, the oxidation effects are difficult to detect.

The summary of DSC/TGA/MS results is shown in Figure 4. First, we focus on a representative SiQD-{111} sample as Figure 4a,b compares their DSC/TGA and MS curves with those of c-SiNCs. Figure 4c then shows the DSC/MS curves for the representative SiQD sample for comparative purposes and plots the derivative of TGA (dTGA). This derivative highlights the portions of the TGA curve where changes occur. Thus, the good correspondence between the DSC and the dTGA curves in Figure 4c demonstrates that the detected exothermic processes are connected with the increase of sample weight (through oxidation). Next, the influence of SFE on the surface reactivity of SiQDs, in particular the dehydrogenation and thermal oxidation of their surface, is discussed separately, see Figure 4d–f. The processes are described using two types of temperature labeling: TGA-based temperature intervals, marking the individual oxidation-related zones, and DSC onset temperatures, which indicate the temperatures at which the release of energy starts. Please note that we opted to classify the zones based on oxidation processes, which do not include dehydrogenation. The latter process is therefore not in close alignment with the chosen boundaries of the zones (see Figure 5).

#### Zone 1 (Relaxation of Surface and Structural Defects)

The changes occurring in Zone 1 in the temperature range 25–180 °C in SiQD-{111} under oxidizing atmosphere are not connected with any weight gain, as is clear from the TGA curve in Figure 4a,c, thus, no oxidation takes place. However, the DSC curve (Figure 4a) features a weak exothermic event with onset temperature at 79 °C (*T*_max_ = 130 °C). As is evident from the measurements under inert atmosphere, a small amount of hydrogen (onset temperature 70 °C) is released in this temperature range, see Figure S13c. Very likely, some hydrogen is released also under oxidizing atmosphere, and it is partially transformed into water before detection while oxygen is consumed, see Figure 4b. The release of hydrogen in Zone 1 is unlikely to originate in the thermal breaking of Si–H_*x*_ bonds, since the temperature is too low.^3,26,38,55,56^ Therefore, the process responsible for the exothermic event occurring in Zone 1 is the relaxation of surface and structural defects,^55^ and or, alternatively, the out-diffusion of H_2_ from interstitial sites within the SiQD lattice.

#### Zone 2 (Backbond Oxidation, Hot Spots, and Dehydrogenation)

A wide range of effects occurs in Zone 2 in the temperature range of 180–450 °C in SiQD-{111} under oxidizing atmosphere: there is a weight gain related to oxidation (Figure 4a, TGA), which is highly exothermic (Figure 4a, DSC) and accompanied by the release of hydrogen, small amounts of SiH_2_ and SiH_3_ radicals and even water (Figure 4b). At the start of Zone 2, backbond oxidation, proceeding primarily through the breakage of the bonds between surface Si atoms, starts to play an important role.^56,57^ However, unexpectedly high amounts of released hydrogen were detected at sample temperatures even below 200 °C, see the MS H_2_ curve in Figure 4b. Typically, higher surface hydrides *Si–H_2/3_ dehydrogenate in the 200–400 °C temperature range in nanocrystalline silicon, whereas stronger monohydrides *Si–H dehydrogenate at even higher temperatures at 400–600 °C, see Figure 4b.^3,26,38,55,56^ Please note that the difference in dissociation temperatures results from activation energies of the dissociation processes, which can substantially vary in surface hydrides with different compositions and even on different surfaces.^58,59^ The breakage of *Si–H bonds in higher hydrides observed here can be explained by the formation of hot-spots within the sample locally at higher temperature as a result of the poor heat conductivity of SiQDs in powder form.^60^ The subsequent exothermic reaction, evident in the DSC curve with the onset at about 240 °C (*T*_max_ = 309 °C) most likely accelerates the process of H release by out-diffusion. It results also in the dehydrogenation of the stronger Si–H bonds in monohydrides, as most hydrogen including that in the monohydrides is released within Zone 2, see the MS H_2_ curve in Figure 4b. In addition to H_2_, the detected water also signifies the release of hydrogen under oxidizing atmosphere, as hydrogen can be partially converted to water before detection. The same measurements carried out under inert atmosphere (Figure S13b) are helpful in revealing the structural changes in the absence of oxidation. Here, the dehydrogenation occurs in a somewhat wider temperature range and its slower progression results from a smaller influence of hot-spots due to the absence of oxidation as an additional exothermic effect promoting H release in any form. The dehydrogenation under inert atmosphere lacking the oxidation-related acceleration is evidenced by the MS H_2_ peak with onset at 150 °C and a side shoulder with onset at 330 °C (Figure S13c). These two peaks are likely connected to the dehydrogenation of higher hydrides and monohydrides, respectively, based on the relative strengths of the two types of bonds.^3,26,38,55,56^ Virtually no water and about twice as much hydrogen is detected under inert atmosphere as compared to the oxidizing ambient because released hydrogen cannot be effectively transformed to water before detection. The amount of energy released under the two atmospheres is comparable up to about 240 °C, above which oxidation leads to a more rapid energy release under oxidizing atmosphere. Similarly, the Si–H_2/3_ species are also not detected under inert atmosphere, implying that their release is driven by surface oxidation. Thus, we clearly show that the co-occurrence of oxidation and dehydrogenation is the driving factor for the highly exothermic processes in Zone 2, being responsible for the sharp DSC peak absent in other types of nanostructured silicon or under inert atmosphere.

#### Zone 3 (Residual Dehydrogenation and Rupture of Agglomerates)

At the beginning of Zone 3, which extends between 450 and 720 °C in the SiQD-{111} sample under oxidizing atmosphere, oxidation progresses through the transformation of separate suboxide fragments into a compact layer, just a few atomic monolayers thick,^38,56,61^ as evidenced by the weight gain (Figure 4a, TGA) and the ongoing consumption of oxygen (Figure 4b). Furthermore, dehydrogenation of the residual monohydride species not yet dehydrogenated due to hot-spots proceeds, as confirmed by the MS H_2_ peak (Figure 4b) and by the broader peak with a gradual increase and onset temperature of 485 °C in the DSC curve (Figure 4a). In addition, the DSC curve shows a sharp and strongly exothermic effect with maximum at 672 °C and a temperature range of 630–700 °C. This effect was observed in the past,^3,55^ and was attributed to the crystallization of Si nanoparticles^55^ or to diffusive oxidation.^3^ We disagree with both these interpretations because (i) our SiQDs are already crystalline, (ii) the energy release in the temperature range from 630 to 700 °C significantly exceeds the amount to be expected from a mere relaxation of Si atoms from a disordered into a more ordered phase, and (iii) diffusive oxidation proceeds at later stages of the heating process, as shown below. A peculiar feature of this process is the detection of high amounts of water and the consumption of oxygen with no detectable release of hydrogen at 675 °C (Figure 4b). Clearly, at temperatures above 600 °C the sample should be free of hydrogen and water. Therefore, we ascribe this effect to the rupture of agglomerates of SiQDs. Si nanocrystals are known to form agglomerates and aggregates strongly bonded via the growing surface oxide, starting at the very early stages of oxidation.^62^ Going through Zone 2, SiQDs first oxidize and agglomerate, which results in the sealing of microvoids in between SiQDs. Then, the dehydrogenation of silicon hydrides occurs throughout Zones 2 and 3. After reaching a critical temperature of 630 °C, the agglomerates are no longer stable as a result of high temperature and the overall stage of oxidation. Therefore, the agglomerates rupture, the remaining hydrogen trapped after dehydrogenation is released and is detected as water after it was oxidized. Moreover, the exposition of the as of yet unoxidized SiQD surfaces results in oxidation (see the peak in the TGA curve in Figure 4a), which is rapid and highly exothermic. Our interpretation is corroborated by measurements under inert atmosphere, where neither the exothermic DSC effect (Figure S13b) nor the detection of higher amounts of hydrogen or water (Figure S13c) occur, because the absence of oxidation did not lead to the formation of agglomerates. Thus, the driving factor behind the second sharp exothermic effect observed during the thermal oxidation of SiQDs is the rupture of oxide-bonded agglomerates and the subsequent oxidation of the newly exposed surfaces. In a nutshell, Zone 3 could be described by thermal cracking with subsequent oxidation of the exposed Si surfaces.

#### Zone 4 (Diffusive Oxidation)

In the last Zone 4, extending between 720 and 1050 °C in the SiQD-{111} sample under oxidizing atmosphere, the oxidation of silicon in SiQDs is completed via diffusive oxidation. This interpretation is confirmed by the continuous rise in TGA, the peak in the DSC signal with onset temperature at 707 °C and *T*_max_ = 892 °C (Figure 4a), the absence of any effects under inert atmosphere (Figure S13) and the continuous consumption of oxygen (Figure 4b). The rupture of agglomerates occurring in the preceding zone facilitates the diffusion of oxygen to the cores of SiQDs and contributes to the completion of the oxidation process at lower temperatures.

### Changes in Thermal Properties with Dominant Faceting

The overall TGA-derived weight gain is very similar regardless of dominant faceting, signifying that the difference in the amount of surface-bonded hydrogen in the three types of samples under study is not significant, at least in terms of its relative weight. The differences in the three samples will be discussed for the individual zones separately.

#### Zone 1

The main difference observed in the other two samples with higher SFE during the surface and defect relaxation stage is the lower DSC onset temperature (around 50 °C, see Figure 4d,e), implying that the energy release begins at lower temperatures. Moreover, in agreement with the increased percentage of less stable higher surface hydrides as shown in Figure 2f, the {001} sample, in contrast to {111}, releases much more hydrogen before reaching Zone 2, see Figure 4f. This effect is especially apparent in the MS curves under inert atmosphere (Figure S14).

#### Zone 2

Within the backbond oxidation and dehydrogenation stage, both the samples with higher SFE released about 30% more energy than {111}, see Figure 4d. The dehydrogenation process at ≈200 °C differ considerably on a per-sample basis, see Figure S14. The least amount of hydrogen is released by the medium-SFE sample {001}, because its much more profound dehydrogenation had occurred already in Zone 1. Also, the side shoulder of the MS H_2_ peak attributed to the dehydrogenation of surface monohydrides is the least evident in the {001} sample, which has the lowest proportion of surface monohydrides, see Figure 2f. The DSC onset temperature of Zone 2 gradually shifts to lower temperatures (Figure 4e) with increasing SFE down to roughly 100 °C at p{110}. This downshift correlates well with the expected lower stability of the higher-SFE samples.

#### Zone 3

In the zone characteristic for residual dehydrogenation and rupture of agglomerates, there are detectable differences in the initial dehydrogenation stage. In good agreement with the composition of surface hydrides (Figure 4d), no hydrogen is released by the {001} sample which has a higher proportion of less stable higher hydrides. On the other hand, the MS H_2_ peak of the p{110} sample predominantly terminated by monohydrides (Figure S14f) still has a detectable tail here, see Figure 4f. The onset temperatures of Zone 3 are almost the same for all three samples, see Figure 4e, because the outermost atomic shells of the QDs, which have already been covered with a monolayer of oxide, are not as sensitive to the silicon surface bonds and dominant faceting as in the initial stage of oxidation. After exceeding 650 °C and the rupture of agglomerates, the most energy is released by the medium-SFE sample {001}, which is a consequence of its most complex composition of surface hydrides getting decomposed. This decomposition causes its highest propensity to agglomerate.

#### Zone 4

The only significant difference between the three samples in the diffusive-oxidation zone is the lowering of both the TGA and the DSC onset temperature and the lowering of the temperature when the oxidation is finished (Figure 4d). This effect results from (i) a lower stability of the higher-SFE QDs and/or (ii) a somewhat smaller size of the more surface-etched, higher-SFE QDs.

We therefore, despite the complicated trends observed during the thermal oxidation of SiQDs, successfully explained the evolution of the DSC/TGA/MS curves in all the SiQD samples based on the existing knowledge of oxidation and dehydrogenation in nanostructured silicon. The key factors driving the processes are differences in SFE and the closely related differences in the composition of surface hydrides. Our description successfully explains even the observed hydrogen released in the highest-SFE partially structurally disordered SiQD-p{110} at relatively higher temperatures when compared to the other two types of samples, which would not be an effect intuitively expected. Thus, these results underline the importance of SFE and dominant faceting in the properties of QDs.

### Dominant Faceting versus Size Dependence

In principle, the differences in surface reactivity could be caused by the slightly different size distributions of the synthesized nanoparticles (see Figure 2c). There are several arguments against this interpretation. A preliminary assessment of the extent to which the size and dominant faceting influence SFE can be based on our calculations from Figure 3b using mean sizes of the individual samples. This analysis shows that, for an ≈5 nm QD, the changes in SFE induced by these two factors are at least comparable (). However, surface reactivity experiments probe the whole size distributions rather than just the mean sizes. Thus, the overlap of the distributions of 0.75 and 0.88 as characterized by the Bhattacharyya coefficient reduces the impact of size even further. Moreover, a closer look at the HRTEM-derived histograms in Figure S6 reveals that the size distribution of the {111}-dominantly faceted sample could be slightly bimodal, which is an additional factor contributing to the overlap of the first two distributions. Importantly, the inherent connection between size and SFE is strictly *monotonous*, see Figure 3b. In contrast to a monotonous trend, in our experiments, the {001}-dominantly faceted middle-SFE (and medium-sized) sample exhibits a detectably nonmonotonously different dynamics of the dehydrogenation process, clearly determined by its dominant faceting rather than size-induced SFE changes, see Figure 4f. Therefore, our experiments justify the proposed assignment of dominant faceting as an important factor in surface reactivity.

### Tuning of Thermal Oxidation in Silicon Quantum Dots

The trends observed during the thermal oxidation process are schematically summarized in Figure 5. This scheme clearly shows how higher SFE leads to the lowering of the end points of the TGA-based temperature intervals for the individual stages of thermal oxidation. The exception to the rule here is, quite intuitively, the Zone-3 growth of a thicker SiO_2_ shell on an already oxidized surface. Moreover, the DSC-based onset temperatures also decrease with increasing SFE with the exception of Zone-3 residual dehydrogenation. However, the subsequent agglomerate-rupture-related energy release is finished at lower temperatures in higher-SFE samples. The release of hydrogen is then influenced by both the SFE and the composition of surface hydrides.

An interesting feature observed in Zone 1 is the relatively high amount of released energy, especially considering DSC measurements under inert atmosphere, see Figure S11c, where no energy can be released from hydrogen. Therefore, we attribute the energy released in this temperature range to the structural reorganization of the surface. In general, structural relaxation in a similar temperature region was described mostly in glassy systems.^63^ However, an analogic phenomenon can occur in Si (or other) QDs. The exothermic structural relaxation results from the increased fluidity or mobility of atoms due to the increase of free volume, which is the difference of an average volume per molecule/atom in the liquid and its van der Waals volume.

Figure 5 provides a guide for the tuning of the thermal properties of SiQDs via dominant faceting. Clearly, ignition temperatures as low as 150 °C are attainable in SiQDs if desired. However, if need be, the energy release in the Zone-2 temperature range can be bypassed by performing slow natural oxidation under ambient conditions. In such an oxidized sample, major energy release would come from the Zone-3 rupture of agglomerates at much higher temperatures >600 °C. This scenario very likely occurred, perhaps inadvertently, in the study of the combustion of amorphous Si nanoparticles,^3^ where, similarly to our measurements, the DSC curves of H-terminated Si nanoparticles showed a major peak at low temperatures around 200 °C. However, ignition of a nanoparticle-based composite happened at much higher temperatures (>700 °C). This discrepancy is likely to have been caused by a thin layer of oxide on silicon nanoparticles in the combustion experiments, formed during the ultrasonication step when the nanocomposites were prepared. It appears that in their^3^ combustion experiments, the silicon nanoparticles were no longer H-terminated and the ignition was initiated by the hydrogen released after the rupture of the agglomerates. This example illustrates how, using a combination of SFE and surface chemistry, the ignition temperature of Si nanoparticles can be tuned from temperatures as low as 150 °C to >700 °C.

Apart from ignition-related properties useful for energetic applications,^3^Figure 5 also hints toward the possibility of using SiQDs for hydrogen storage since hydrogen release can be achieved under various conditions. Even though our findings represent fundamental characteristics of this material, the main motivation driving this research is the potential of having resource-efficient^21^ material with inherently low toxicity^20^ since silicon is the second most abundant element in the Earth’s crust and a trace element in humans. Unlike other traditional energy-storage materials, silicon is so biocompatible that high-quality SiQDs can even be synthesized using rice husks, which is agricultural waste, as the source of silicon.^64^ Naturally, countless other issues need to be resolved before a potential production roll-out, including the reversibility of the oxidation reaction leading to highly stable products. Whereas complete oxidation including the Zone-4 diffusive stage is very likely irreversible because the QDs are destroyed, the reversibility of the surface backbond oxidation is currently being tackled in silicon-air batteries.^21^ Interestingly, plasma synthesis of silicon nanoparticles itself has already been commercialized. Slightly larger silicon nanoparticles (∼12 nm) formulated into a screen printable ink are already available as a selective emitter layer in silicon solar cells by Innovalight Corporation.^65^ Besides, the utilization of silicon is considered in Li-based batteries,^66,67^ partially because Si nanoparticles and QDs can accommodate expansion.^68^

While thermal oxidation was chosen as the studied process here because SiQDs are highly prone to oxidation and readily oxidize when exposed an environment containing oxygen species, it is also possible to tune the energy-storage properties by changing the reactive environment. However, such a study is beyond the scope of this article.

### Mechanism of Ignition

The combined measurements allow us to identify the processes responsible for the relatively low-temperature ignition of Si nanoparticles reported in Figures 1 and S8. The initially proposed possibility of the combustion being linked to trapped hydrogen was proven wrong by experiments using the “fresh” and “aged” varieties of samples. These experiments demonstrate that the amount of trapped, not chemically bonded hydrogen is negligible. Thus, the low-temperature combustion is linked almost exclusively to the highly exothermic hot-spot-driven processes occurring at the beginning of Zone 2. Several factors influence the observed ignition process. First, in general, SiQDs are known to have low thermal conductivity.^69^ Based on calculations presented in Ref (19) the effective thermal conductivity *k*_eff_ of our samples (porosity 0.77) is roughly 0.1 , which is much closer to air ( at 30–200 °C) than to silicon ( at 30–200 °C). These conditions favor the backbond-oxidation-driven formation of hot-spots locally at much higher temperatures, as locally, temperatures around 400 °C can be reached solely as a result of absorption of light.^19^ Such temperatures are well within the temperature range of the typical dehydrogenation of silicon hydride bonds, see Figure 4b. Next, the oxidation of hydrogen in contact with air results in a gaseous reaction product diffusing away (vs SiO_2_ staying put as a solid), and a considerable amount of thermal energy is being passed on to neighboring Si atoms as a result of dehydrogenation. Thus, the released hydrogen provides sufficient energy to drive further dehydrogenation and, finally, also the diffusive oxidation of the whole volume of the sample.

The ignition temperature is lower in amorphous nanoparticles, see Figure 1d, as, in accordance with the trends reported in Figure 5, the thermal oxidation processes start earlier in less structurally ordered nanoparticles. In addition, amorphous Si nanoparticles can store and thus also release higher amounts of H when heated. Since H is much more reactive in bonding with oxygen as compared to Si, a catalytic Si oxidation due to local heat transfer from H undergoing an oxyhydrogen reaction lowers the combustion temperature of amorphous Si nanoparticles.

On the contrary, when the combustion process is initiated by light absorption rather than by heating, light is absorbed primarily by nanoparticles, which are insulated from one another by air. Here, the key factor driving the combustion process is the dissipation of heat from the nanoparticles to the surrounding air. Therefore, this process is unlikely to be strongly dependent on the degree of structural order, instead, a difference between fully amorphous and structurally ordered nanoparticles can be expected. This expectation is confirmed by the trends observed in Figure 1d,f.

No ignition analoguous to that described in Figure 1 was observed in the DSC experiments. The lack of this effect can be caused by (i) a lower oxygen content in the oxidizing atmosphere during the DSC/TGA/MS measurements and (ii) faster heating rates in the ignition experiments, which lead to more pronounced hot-spots and (iii) the pumping present in the DSC/TGA/MS measurements, which causes the outflow of the reaction byproduct from the reactor so that it could be analyzed.

### Other Considerations

As a result, surface properties, thermal properties and the overall stability of SiQDs can be tuned by SFE. In this regard, synthesis in nonthermal plasma could be the ideal method for SFE tuning. The two other most frequent competitive methods capable of producing SiQDs are electrochemical etching^71^ and thermal annealing of Si-rich silicon-oxide matrices.^72,73^ During electrochemical etching, SiQDs are “extracted” from an initially ideally crystalline wafer with a defined crystalline orientation through an HF-based wet electrolytic etching process. In the thermal annealing approach, a silicon-rich glass with a suitable composition undergoes thermal annealing at high temperatures >1000 °C, which induces the diffusion of silicon atoms, leading to the formation of QDs. Then, the oxide matrix is removed by HF etching. Neither of these methods seems promising in tuning the surface properties of the produced QDs: etching of a monocrystal in a predefined crystalline direction is unlikely to lead to QDs with varying surface faceting and the degrees of freedom of the migration of ions inside a solid amorphous matrix is in principle significantly limited by the matrix itself. Thus, while dominant faceting in SiQDs can be tuned, it is possible that only some methods can provide such tuning abilities, with the synthesis in nonthermal plasma being an ideal candidate.

One more interesting observation we want to discuss pertains to the highest-SFE sample SiQD-p{110}. Based on FTIR spectroscopy, we argue that the surface of this sample seems less ordered than in the other SiQD samples studied here, most likely as a result of the highest SFE. However, it still contains a high proportion of surface monohydrides and its surface geometry is analogous to a {110} faceted Si surface. A model of an SiQD being a heterostructure comprising a structurally ordered core and an “amorphized Si shell” has been proposed in SiQDs fabricated by thermal annealing.^46,73^ Here, we prefer to use the term “degree of structural order” rather than the bulk-based dichotomy of crystalline vs amorphous materials, as the question of crystalline order in QDs is more complex.^74^ Moreover, we believe that the difference in the size distribution as determined from coherently diffracting domains in XRD and using HRTEM^46^ reflects the layer in a QD affected by surface reconstruction, which does not necessarily need to be amorphous. This quantity is closely related to the degree of structural order of the surface which we describe using SFE. Leaving this issue aside, the obvious question which comes to mind is, if and how much the proposed amorphized shell is linked to the HF-etching step of the fabrication process in the thermally annealed SiQDs. It is entirely plausible that the interaction of the surface of synthesized QDs, whatever its initial structural properties might be, with a highly corrosive agent might change the initial surface structure to a less ordered state. For planar Si surfaces such as crystalline Si wafers, such increased disorder reveals itself by an increased surface roughness. Thus, the structural properties of the surface, as discussed here and in Refs (73 and 75) can be highly dependent on the fabrication method and sample history. However, at this point, we leave this hypothesis unproven, pointing to further research efforts.

## Conclusions

We introduced the concept of dominant faceting as macroscopically detectable preferential structural order of the surface of QDs, present even without evident shape control on the microscopic scale. Dominant faceting describes a state in which a certain type of facet dominates at the surface of highly polyhedral quasi-spherical QD, determining the QD’s surface reactivity regardless of the concrete spatial distribution of the facets on the surface. We tentatively propose that local strain induced by surface atoms on the facets is responsible for the changes in reactivity, as supported by classical force field simulations. Furthermore, we showed that dominant faceting can be applied to tune the thermodynamical properties of QDs, such as their stability or surface reactivity, using SiQDs as a case-study material. In particular, we derived analytical expressions for surface free energy for three model geometries of SiQDs differing in dominant faceting. Next, we established a link between the measured surface properties of synthesized SiQDs with the surface free energy of the idealized SiQD models and show that this association successfully predicts the lowering of onset temperatures of different exothermic processes during thermal oxidation. We discussed that SFE tuning can potentially be dependent on the fabrication method, with synthesis in nonthermal plasma being able to attain a wide range of SFE in SiQDs. Moreover, the energy released during thermal oxidation was shown to be very close to theoretical limits, confirming that full oxidation of all the contained silicon material is achieved. This finding underpins the potential of silicon as an energy-storage material. Next, using combined DSC, TGA and MS measurements, we identified the processes responsible for the release of energy during thermal oxidation of SiQDs. Here, especially the release of surface-bonded hydrogen was shown to be tunable in a wide range of temperatures by surface properties. In addition to introducing dominant faceting, our experiments provide a detailed guide for using SiQDs as an energy-storage or thermal material, by showing that both low-temperature ignition (≈ 200 °C) and higher-temperature energy release (≈ 650 °C) can be obtained by tuning their SFE.

## Methods

This section lists only a brief overview of the applied methods, see Supporting Information for a more detailed description.

### Synthesis of Nanoparticles

SiQDs were synthesized using a noncommercial flow-through reactor in nonthermal plasma and collected onto glass covers for microscopy. Synthesis parameters for the individual samples are listed in Table 1. The commercial oxidized Si nanoparticles (c-SiNCs) were purchased from PlasmaChem GmbH. The partially hydrogen-terminated commercial Si nanoparticles (H-c-SiNCs) were etched using HF.

### Study of Ignition Conditions

In the heat-initiated ignition, the sample was placed on a hot plate on top of an aluminum foil and temperature was increased stepwise from 50 °C until ignition. The light-initiated ignition was realized using a standard 405 nm laser pointer. The measurements were repeated several times to obtain standard deviation of the ignition temperature.

### Structural and Chemical Characterization

X-ray diffraction (XRD) measurements were performed on a Rigaku SmartLab diffractometer equipped with a 9 kW rotating anode Cu source (wavelength K_α1_ λ = 0.154056 nm) in a semi focusing Bragg–Brentano geometry. Diffracted intensity was counted by a 2D hybrid pixel single photon counting HyPix3000 detector.

The ordered mesoporous structure was determined from small-angle X-ray scattering (SAXS) using Xenocs Xeuss 2.0 SAXS instrument equipped with the Mo K_α_ (λ = 0.07107 nm) radiation X-ray microfocus sources, toroidal X-ray mirror and scatter-less slits producing collimating parallel beam point focus, and a Pilatus 200k (Dectris) hybrid pixel single-photon counting detector.

High-resolution transmission electron microscopy (HRTEM) measurements were carried out using EFTEM Jeol 2200 FS operated at 200 kV. Surface of HRTEM analyzed SiQDs was dodecyl terminated using the thermal hydrosilylation method. The acquired HRTEM images were analyzed manually.

The Fourier-transform infrared (FTIR) absorption analysis was realized using Nicolet iS50 FT-IR microscope (Thermo Scientific). The measurements were performed in the attenuated total reflectance regime using a monocrystalline diamond crystal. The Raman spectra measurements were realized using microspectrometer Renishaw inVia Reflex equipped with a HeCd continuous laser (Kimmon Dual Wavelength HeCd) using the excitation wavelength of 442 nm. We cannot rule out that some of the observed shifts in the 520 cm^–1^ are caused by sample heating.

### Determination of the Ratios of Surface Hydrides

Based on published analysis,^26^ we used one peak for the SiH_3_ surface stretching mode (2140 cm^–1^), two peaks for the stretching modes of SiH_2_ (2100 and 2117 cm^–1^) and two peaks for the stretching modes of SiH (2070 and 2085 cm^–1^). The ratio of the integrated peaks was used to determine the surface hydride composition. Each data point of SiH_*x*_ composition represents at least six independent measurements.

### Classical Force Field Simulations of Strain

The classical force field simulations were conducted with homemade code FireCore.^54^

### Calorimetry

The gravimetric energy density was determined using a commercial Combustion compensated jacket calorimeter Parr, Model 1351, (Parr Instr. Comp., Moline, IL, USA), equipped with an oxygen bomb Parr 1108.

### DSC/TGA/MS

All samples were measured by nonisothermal DSC-TG analysis using the Setaram Themys 2400 simultaneous thermal analyzer, which was coupled by a quartz capillary with the Pfeiffer Vacuum OmniStarTM GSD320 mass spectrometer. Data from all performed analyses (DSC-TGA-MS) were processed by the Calisto Processing software. Each experiment was repeated 2–3 × for every sample, repeated experiments led to very similar trends.

## Data Availability

The associated datasets are available at: https://doi.org/10.57680/asep.0603089.

## Supporting Information Available

Derivation of the absolute SFE as a function of QD faceting and size, details of the applied methods, additional MS/DSC/TGA and other analyses, and photographs of samples (PDF)
Example of light-initiated ignition (MP4)
