# Supplementary material for: Engineering the Thermal and Energy-Storage Properties in Quantum Dots Using Dominant Faceting: The Case Study of Silicon
Source: ACS Nano. 2025 Jan 6;19(2):2196–212. doi: 10.1021/acsnano.4c11376 (PMC11760162; doi:10.1021/acsnano.4c11376)
Supplement: Supplementary file 2 — nn4c11376_si_002.pdf [file nn4c11376_si_002.pdf]

Supporting Information: Engineering the Thermal and Energy-Storage Properties in Quantum Dots using Dominant Faceting: the Case Study of Silicon

Pavel Galář,[†] Jakub Kopenec,[†] Robert Král,[†] Filip Matějka,[†] Petra Zemenová,[†] Milan Dopita,[‡] Prokop Hapala,[†] Dirk König,[¶,§,‖] Pavel Vrbka,[⊥] and Kateřina Kůsová[*,†]

†*Institute of Physics of the CAS, v.v.i., Cukrovarnická 10, 162 00 Prague 6, Czechia*

‡ *Faculty of Mathematics and Physics, Charles University, Ke Karlovu 3, 121 16 Praha 2, Czechia*

¶ *Integrated Materials Design Lab, The Australian National University, Canberra, ACT 2601, Australia*

§ *Department of Applied Mathematics, Research School of Physics, The Australian National University, Canberra, ACT 2601, Australia*

‖ *Institute of Semiconductor Electronics (IHT), RWTH Aachen University, 52074 Aachen, Germany*

⊥ *University of Chemistry and Technology, Technická 5, 166 28 Praha 6, Czechia*

E-mail: kusova@fzu.cz

1 Derivation of surface free energies (SFEs) for all three QD Topologies

Extending our previous analysis,[S1] we derive expressions for the facet-specific surface areas per QD type as a function of their size. We use a spherical approximation for QD sizes, as otherwise, different QD topologies cannot be compared directly. With the atomic volume per QD atom V_{atom} (Si: $V_{\mathrm{atom}} = 0.020023\ \mathrm{nm}^3$), we obtain a gauge for QD (or nanocrystal; NC) sizes simply by

$$d_{\mathrm{NC}}[i] \;=\; \sqrt[3]{\frac{6}{\pi} V_{\mathrm{atom}} N_{\mathrm{NC}}[i]} \qquad \text{(S1)}$$

The run index i is the defining parameter for QD sizes[S1] and serves to the same purpose here. The number of Si atoms forming the QD is given by $N_{\mathrm{NC}}[i]$.[S1]

Next, we derive the facet-specific surface areas. Due to the high symmetry of the fcc lattice and QD topologies, we merely need to derive a characteristic edge length $a[i]$ and plug this length into the surface expression per facet type and QD topology. Starting with the {111}-dominated quatrodecahedral QD type, we see that its surface consists of eight regular hexagons with {111}-orientation, and six squares with {001}-orientation, *cf.* Fig. S1. Regular hexagons are composed of six equilateral triangles, a relation we will put to good use below.

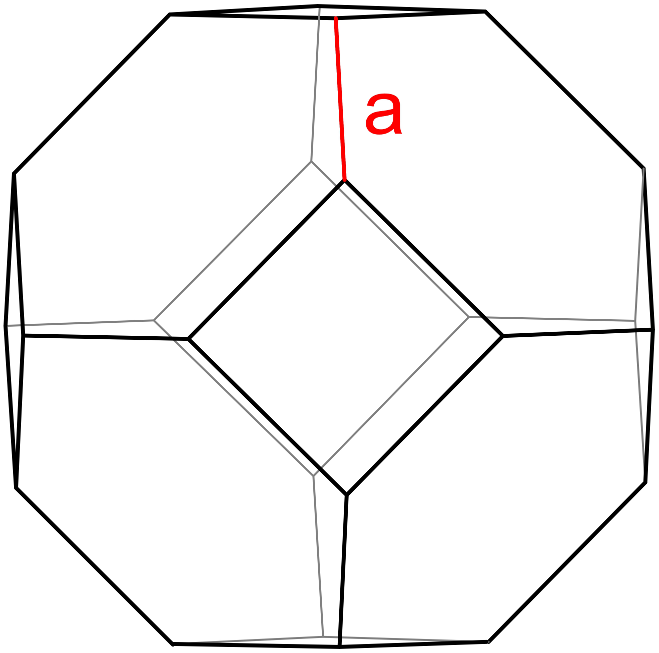

Figure S1: Frame graph of regular {001}-dominated quatrodecahedron with six {001}-faceted squares and eight {111}-faceted regular hexagons. The side length $a = a^{\mathrm{q.111}}$ describes all facet areas.

When evaluating the side length a of {001}-dominated quatrodecahedra, we arrive at the following values. We see immediately that

Table S1: Values for the number series of $a^{\mathrm{q.111}}[i]$, $N_{\mathrm{NC}}^{\mathrm{q.111}}[i]$, and $d_{\mathrm{NC}}[i]$ for run index $i = 1 \ldots 5$. The characteristic length is given in unit cell lengths a_{uc}.

i	1	2	3	4	5
$a^{\mathrm{q.111}}[i]$ $[a_{\mathrm{uc}}]$	$\frac{1}{2}\sqrt{2}$	$\frac{2}{2}\sqrt{2}$	$\frac{3}{2}\sqrt{2}$	$\frac{4}{2}\sqrt{2}$	$\frac{5}{2}\sqrt{2}$
$N_{\mathrm{NC}}^{\mathrm{q.111}}[i]$	66	377	1126	2505	4706
$d_{\mathrm{NC}}[i]$ [nm]	1.36	2.43	3.51	4.58	5.65

$$a^{\mathrm{q.111}}[i] \;=\; \frac{a_{\mathrm{uc}}}{\sqrt{2}}\, i\ , \tag{S2}$$

whereby a_{uc} stands for the unit cell length of the lattice considered. Next, we use $a^{\mathrm{q.111}}[i]$ to calculate the facet area per surface orientation. Each of the eight regular {111}-hexagons can be decomposed into six equilateral triangles with side length $a^{\mathrm{q.111}}[i]$. The area of an equilateral triangle is

$$A_{\triangle}^{\mathrm{q.111}}[i] \;=\; \frac{\sqrt{3}}{4}\left(a^{\mathrm{q.111}}[i]\right)^2 , \tag{S3}$$

As six $A_{\triangle}^{\mathrm{q.111}}[i]$ describe the area of one hexagonal {111}-facet, and we have eight of such facets per Si QD, we obtain for the total area with {111}-orientation

$$A_{111}^{\mathrm{q.111}}[i] = 48\, A_{\triangle}^{\mathrm{q.111}}[i] \;=\; 6\sqrt{3}\, a_{\mathrm{uc}}^2\, i^2 \tag{S4}$$

Calculating the total area of the {001}-oriented facets is straightforward via the area of such squares being $A_{\square}^{\mathrm{q.001}}[i] = (a^{\mathrm{q.001}}[i])^2 = 1/2\, a_{\mathrm{uc}}^2\, i^2$. With six of such {001}-squares per Si QD, we get

$$A_{001}^{\mathrm{q.111}}[i] = 6\, A_{\square}^{\mathrm{q.001}}[i] \;=\; 3\, a_{\mathrm{uc}}^2\, i^2 \tag{S5}$$

for the total area of all {001}-facets. The total surface area of the Si QD follows straight from summing up both orientation-specific area values.

Fig. S2 shows the shape of the {001}-dominated quatrodecahedra QD class. The characteristic length of the {001}-dominated quatrodecahedron is described in Table S2. Again,

Table S2: Values for the number series of $a^{\mathrm{q.001}}[i]$, $N_{\mathrm{NC}}^{\mathrm{q.001}}[i]$, and $d_{\mathrm{NC}}[i]$ for run index $i = 1 \ldots 5$. The characteristic length is given in unit cell lengths a_{uc}.

i	1	2	3	4	5
$a^{\mathrm{q.001}}[i]$ $[a_{\mathrm{uc}}]$	$\frac{4}{2}\sqrt{2}$	$\frac{5}{2}\sqrt{2}$	$\frac{6}{2}\sqrt{2}$	$\frac{7}{2}\sqrt{2}$	$\frac{8}{2}\sqrt{2}$
$N_{\mathrm{NC}}^{\mathrm{q.001}}[i]$	549	1021	1707	2647	3881
$d_{\mathrm{NC}}[i]$ [nm]	2.76	3.39	4.03	4.66	5.30

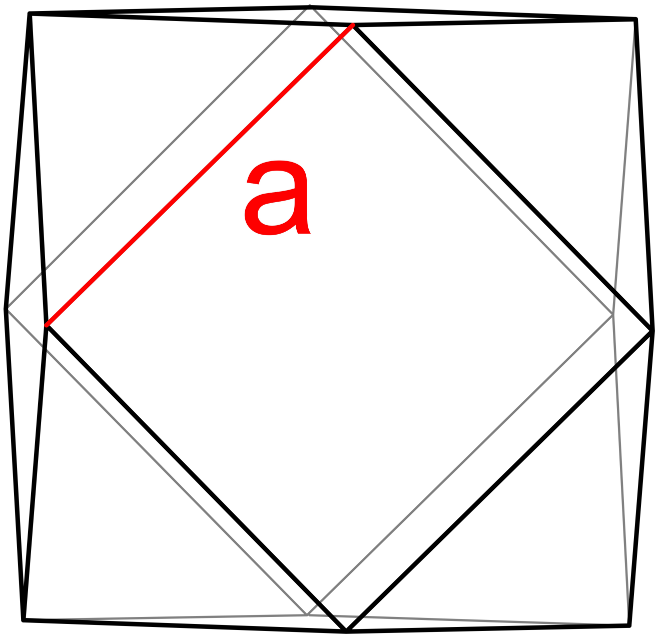

Figure S2: Frame graph of regular {001}-dominated quatrodecahedron with six {001}-faceted squares and eight {111}-faceted regular hexagons. The side length $a = a^{\mathrm{q.001}}[i]$ describes all facet areas.

it is straightforward to see that

$$a^{\mathrm{q.001}}[i] \quad = \quad \frac{a_{\mathrm{uc}}}{\sqrt{2}}\,(i+3)\;, \tag{S6}$$

For the facets of {001}-oriented dodecahedral Si QDs, we have eight equilateral triangles with {111} orientation, and six squares with {001} orientation. In analogy to {111}-dominated quatrodecahedra, we obtain

$$A_{111}^{\mathrm{q.001}}[i] = 8\frac{\sqrt{3}}{4}\left(a^{\mathrm{q.001}}[i]\right)^2 \quad = \quad \sqrt{3}\,a_{\mathrm{uc}}^2\,(i+3)^2 \tag{S7}$$

Calculating the total area of the {001}-oriented facets is once again straightforward via the area of such squares being $A_{\square}^{\mathrm{q.001}}[i] = (a^{\mathrm{q.001}}[i])^2 = 1/2\,a_{\mathrm{uc}}^2\,(i+3)^2$. With six of such {001}-squares per Si QD, we get

$$A_{001}^{\mathrm{q.001}}[i] = 6\,A_{\square}^{\mathrm{q.001}}[i] \quad = \quad 3\,a_{\mathrm{uc}}^2\,(i+3)^2 \tag{S8}$$

for the total area of all {001}-facets. The total surface area of the Si QD again follows straight from summing up both orientation-specific area values.

The final QD topology is given by a regular dodecahedron with exclusive {110}-faceting and characteristic length $a^{\mathrm{dod}}[i]$. This QD type is described by an *even* and an *odd* series.[S1] Fig. S3 shows the shape of the {110} dodecahedral QD class. We first evaluate the *odd* series and look at the evolution of its characteristic length $a^{\mathrm{dod,\,odd}}[i]$: We see that

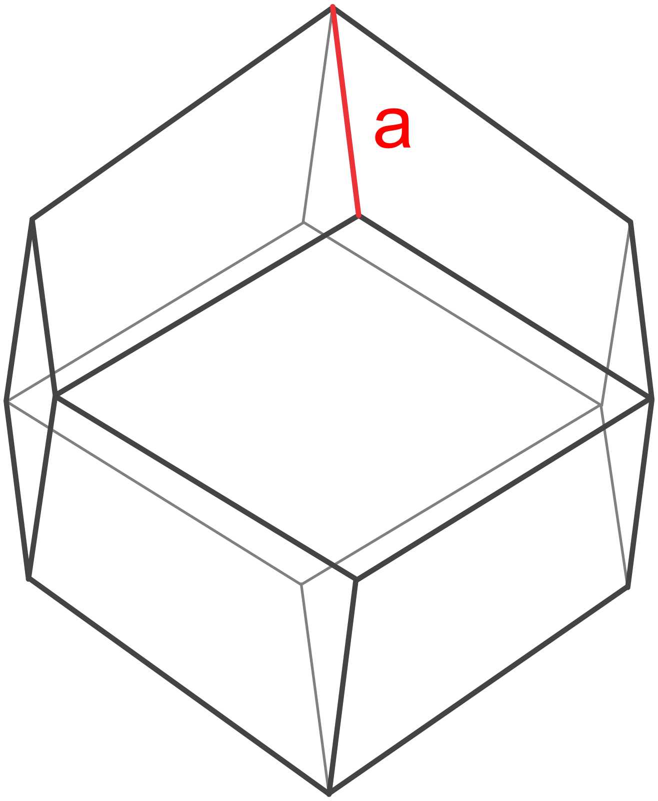

Figure S3: Frame graph of regular {110} dodecahedron featuring twelve {110}-faceted rhomboids. The side length $a = a^{\text{dod}}[i]$ describes all facet areas.

Table S3: Values for dodecahedral NCs, *odd* number series $a^{\text{dod, odd}}[i]$, $N_{\text{NC}}^{\text{dod, odd}}[i]$, and $d_{\text{NC}}[i]$ for run index $i = 1 \ldots 5$. The characteristic length is given in unit cell lengths a_{uc}. Since the calculation of $a^{\text{dod, odd}}[i]$ is more complex, we calculate this length by gauging its extension in horizontal direction ($\langle 110 \rangle$ vector class; $a_{\leftrightarrow}^{\text{dod, odd}}[i]$) and in vertical direction ($\langle 001 \rangle$ vector class; $a_{\updownarrow}^{\text{dod, odd}}[i]$), see also to Fig. 6 in Ref. (S1), and use Pythagoras' theorem to arrive at $a^{\text{dod, odd}}[i]$.

i	1	2	3	4	5
$a_{\leftrightarrow}^{\text{dod, odd}}[i]$ $[a_{\text{uc}}]$	$\frac{5}{4}\sqrt{2}$	$\frac{9}{4}\sqrt{2}$	$\frac{13}{4}\sqrt{2}$	$\frac{17}{4}\sqrt{2}$	$\frac{21}{4}\sqrt{2}$
$a_{\updownarrow}^{\text{dod, odd}}[i]$ $[a_{\text{uc}}]$	$\frac{5}{4}$	$\frac{9}{4}$	$\frac{13}{4}$	$\frac{17}{4}$	$\frac{21}{4}$
$N_{\text{NC}}^{\text{dod, odd}}[i]$	338	1722	4930	10730	19890
$d_{\text{NC}}[i]$ [nm]	2.35	4.04	5.73	7.43	9.13

$$a^{\mathrm{dod,\,odd}}_{\leftrightarrow}[i] \;=\; a_{\mathrm{uc}}\sqrt{2}\,\left(i+\frac{1}{4}\right) \text{ and} \tag{S9}$$
$$a^{\mathrm{dod,\,odd}}_{\updownarrow}[i] \;=\; a_{\mathrm{uc}}\,\left(i+\frac{1}{4}\right)\;.$$

Using Pythagoras' theorem with $a^{\mathrm{dod,\,odd}}_{\leftrightarrow}[i]$ and $a^{\mathrm{dod,\,odd}}_{\updownarrow}$, we obtain

$$a^{\mathrm{dod,\,odd}}[i] \;=\; a_{\mathrm{uc}}\sqrt{3}\,\left(i+\frac{1}{4}\right)\;. \tag{S10}$$

The surface of a regular dodecahedron is given by[S2]

$$A^{\mathrm{dod}} \;=\; 12\,\left[\frac{2\sqrt{2}}{3}\,a^2\right]\;, \tag{S11}$$

whereby the expression in square brackets describes the area of one rhombus. We thus get

$$A^{\mathrm{dod,\,odd}}[i] \;=\; 24\,\sqrt{2}\,a^2_{\mathrm{uc}}\,\left(i+\frac{1}{4}\right)^2\;. \tag{S12}$$

For the *even* series of regular $\{110\}$-faceted dodecahedra,[S1] we follow the derivation above with slight modifications. Again, we first look at the evolution its characteristic length $a^{\mathrm{dod,\,even}}[i]$: We see that

Table S4: Values for dodecahedral NCs, *even* number series $a^{\mathrm{dod,\,even}}[i]$, $N^{\mathrm{dod,\,even}}_{\mathrm{NC}}[i]$, and $d_{\mathrm{NC}}[i]$ for run index $i = 1 \ldots 5$. The characteristic length is given in unit cell lengths a_{uc}. Since the calculation of $a^{\mathrm{dod,\,even}}[i]$ is more complex, we calculate this length by gauging its extension in horizontal direction ($\langle 110\rangle$ vector class; $a^{\mathrm{dod,\,even}}_{\leftrightarrow}[i]$) and in vertical direction ($\langle 001\rangle$ vector class; $a^{\mathrm{dod,\,even}}_{\updownarrow}[i]$), see also to Fig. 6 in Ref. (S1), and use Pythagoras' theorem to arrive at $a^{\mathrm{dod,\,even}}[i]$.

i	1	2	3	4	5
$a^{\mathrm{dod,\,even}}_{\leftrightarrow}[i]$ [a_{uc}]	$\frac{7}{4}\sqrt{2}$	$\frac{11}{4}\sqrt{2}$	$\frac{15}{4}\sqrt{2}$	$\frac{19}{4}\sqrt{2}$	$\frac{23}{4}\sqrt{2}$
$a^{\mathrm{dod,\,even}}_{\updownarrow}[i]$ [a_{uc}]	$\frac{7}{4}$	$\frac{11}{4}$	$\frac{15}{4}$	$\frac{19}{4}$	$\frac{23}{4}$
$N^{\mathrm{dod,\,even}}_{\mathrm{NC}}[i]$	848	3048	7456	14840	25968
$d_{\mathrm{NC}}[i]$ [nm]	3.19	4.88	6.58	8.28	9.98

$$a^{\mathrm{dod,\,even}}_{\leftrightarrow}[i] \;=\; a_{\mathrm{uc}}\sqrt{2}\,\left(i+\frac{3}{4}\right) \text{ and} \tag{S13}$$
$$a^{\mathrm{dod,\,even}}_{\updownarrow}[i] \;=\; a_{\mathrm{uc}}\,\left(i+\frac{3}{4}\right)\;.$$

Using Pythagoras' theorem with $a^{\mathrm{dod,\,even}}_{\leftrightarrow}[i]$ and $a^{\mathrm{dod,\,even}}_{\updownarrow}$, we obtain

$$a^{\mathrm{dod,\,even}}[i] \;=\; a_{\mathrm{uc}}\sqrt{3}\,\left(i+\frac{3}{4}\right)\;. \tag{S14}$$

Finally, the total surface of the {110}-faceted regular dodecahedra of the *even* series is given by

$$A^{\text{dod, even}}[i] \;=\; 24\sqrt{2}\,a_{\text{uc}}^2 \left(i + \frac{3}{4}\right)^2 \,. \tag{S15}$$

.

For assessing the SFE of a specific QD type, we simply form the product of the facet-specific SFE area density $D_{\text{SFE}}^{\{xyz\}}$ and the surface partition with the same facet orientation A_{xyz}. The total SFE per QD is then represented by the sum of all facet-specific expressions: total SFE per QD $= \sum_{xyz} D_{\text{SFE}}^{\{xyz\}} A_{xyz}$. Eaglesham *et al.*[S3] obtained SFE area densities from cleaving c-Si wafers, yielding $D_{\text{SFE}}^{\{001\}} = 1.36$ J/m^2, $D_{\text{SFE}}^{\{111\}} = 1.23$ J/m^2, and $D_{\text{SFE}}^{\{110\}} = 1.43$ J/m^2. The values obtained for all three QD topologies are shown in the inset of Fig 3b in the main text.

Due to the different QD sizes per QD type as given by the analytic descriptions,[S1] a direct comparison of Si QD types is not possible. Moreover, for regular {110}-oriented dodecahedra, we have an *odd* and *even* series which cannot be treated on the same analytic footing. Therefore, analytic least square residual fits are used to the data points of the respective SFE per QD type to describe the SFE for any arbitrary d_{NC} so that a direct calculation of the SFE difference ΔSFE can be obtained. Fig. S4 shows the data points of the SFE with the respective fit for each QD type. Such fits are then used in the main graph of Fig. 3b in the main text.

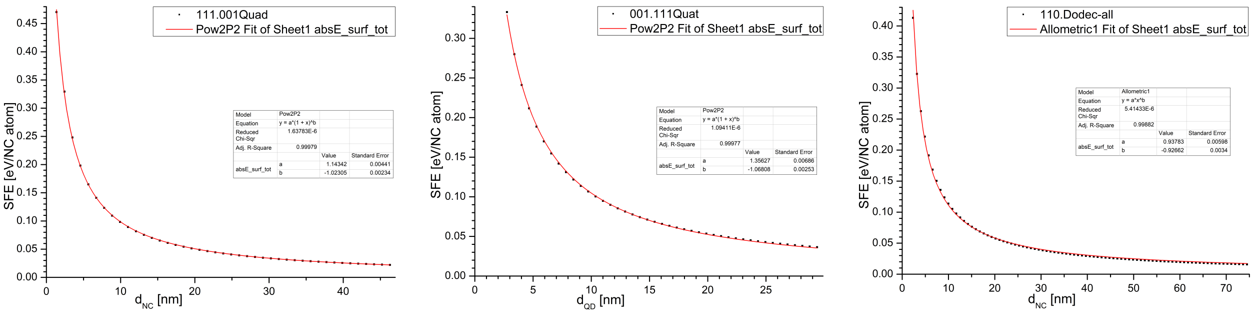

(a) **(b)** **(c)**

Figure S4: Analytic least square residual fits for the total SFE of **(a)** {111}-dominated quatrodecahedral QDs, of **(b)** {001}-dominated quatrodecahedral QDs, and of **(c)** {110} dodecahedral QDs.

2 Methods

2.1 Synthesis of nanoparticles

SiQDs were synthesized using a non-commercial flow-through reactor in non-thermal plasma at low-pressure. The system is based on the system introduced by Kortshagen et al.[S4] The reactor is made up of a one-meter-long glass tube with an inner diameter of 0.8 cm with

attached with copper planar electrodes (5 cm × 12 cm). The plasma is generated by the radiofrequency power source (Coaxial Power Systems, 0−600 W) operating at 13.56 MHz, the output source power ranged from 30 to 150 W. Silane diluted in argon (1 % in argon, Linde, Ar 5.6, SiH_4 5.0, UN1954) and hydrogen (H_2 7.0, Linde, UN1049) were used as the synthesis gases. Low pressure was provided by dry scroll pump (Edwards XDS35i). The pressure in standby is within 10 Pa, in synthesis mode pressures are lower than 500 Pa. Synthesis parameters for the individual samples are listed in Table 1. The synthesized nanoparticles were collected onto glass covers for microscopy 20×20 mm^2, 0.15 mm thickness, Hirschmann, art. n. 8000103 at the bottom of the apparatus.

The commercial oxidized Si nanoparticles (c-SiNCs) were purchased from PlasmaChem GmbH. For the partially hydrogen-terminated commercial Si nanoparticles (H-c-SiNCs), the volume equivalent of 0.5 ml of the c-SiNCs powder was mixed with 5 ml of 99 % ethanol, placed to a teflon bottle and 10 ml of 50 % HF (Penta) was added. The mixture was magnetically stirred at 180 rpm for 2 hours to remove the surface oxide. To remove HF, c-SiNCs were twice centrifugated from the liquid (1.5 krpm, 2 min), most of the liquid was pipetted out and ethanol was added. Additional two purification steps were carried out using n-hexane as the diluting liquid.

2.2 Study of ignition conditions

In the ignition experiments, samples were left on the cover glasses on which they were collected. In the heat-initiated ignition, the sample was placed on IKA RCT basic hotplate (max temperature 320 ℃) on top of an aluminum foil. Temperature was increased stepwise from 50 ℃ until ignition. The actual temperature of the hotplate was measured using Multimeter Voltcraft MT-52 with a probe attached to the aluminum foil. The light-initiated ignition was realized using a standard 405 nm laser pointer (50 mW). The laser pointer was firmly attached to the holder in front of a converging lens (with a radius of 25.4 mm and a focal length of 100 mm). The laser pointer was activated, and the sample was gradually advanced towards the focal point, thus increasing the light intensity on the surface of the sample. At the ignition point, the illuminated area was measured, and the light intensity was calculated. The measurements were repeated several times to obtain standard deviation of the ignition temperature.

2.3 Structural and chemical characterization

X-ray diffraction (XRD) measurements were performed on a Rigaku SmartLab diffractometer equipped with a 9 kW rotating anode Cu source (wavelength $K_{\alpha1}$ $\lambda = 0.154056$ nm) in a semi focusing Bragg-Brentano geometry. The diffractometer was equipped with Johansson monochromator in primary beam producing pure $K_{\alpha1}$ radiation, automatic variable slits, ensuring constant irradiated area of the sample over a whole range of measured diffraction angles and a set of 5° Soller slits eliminating axial divergence in both primary and diffracted beam. Diffracted intensity was counted by a 2D hybrid pixel single photon counting HyPix3000 detector. The diffraction patterns were acquired for diffraction angles 2θ in the range of $20 - 140°$, with a step size $\Delta 2\theta = 0.01°$ and measurement speed of $°.\mathrm{min}^{-1}$.

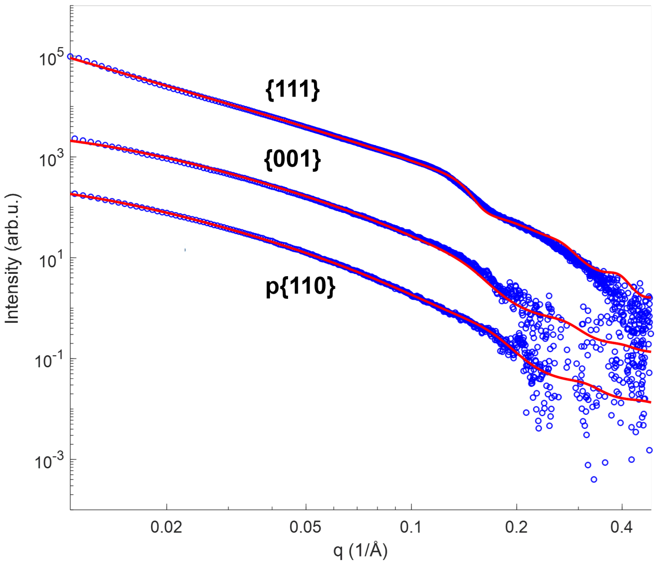

Figure S5: Measured data (blue circles) and fits (red curves) of SAXS data of SiQDs under study, the labelling is explained in the main text (Table 1).

The ordered mesoporous structure was determined from small-angle X-ray scattering (SAXS) using Xenocs Xeuss 2.0 SAXS instrument equipped with the Mo K_α ($\lambda = 0.07107$ nm) radiation X-ray micro-focus sources, toroidal X-ray mirror and scatter-less slits producing collimating parallel beam point focus, and a Pilatus 200k (Dectris) hybrid pixel single-photon counting detector. Scattering vector magnitude q was calculated as $q = (4\pi/\lambda)\sin\theta$, where λ is the wavelength and 2θ is the scattering angle. Calibration of the primary beam position and distances from a sample to the detector was performed using the AgBehenate powder sample. Measured 2D SAXS images were azimuthally integrated into 1D SAXS curves. The scattering from the sample holder (Kapton foil) was subtracted from measured data. The measured data fitting was performed using the SasView computer program.

High-resolution transmission electron microscopy (HRTEM) measurements were carried out using EFTEM Jeol 2200 FS. The field emission gun microscope (ZrO/W FEG) was operated at 200 kV. The images were taken in TEM mode with up to 1,200,000 magnification. The samples were prepared by the application of 7 µl of SiQD dispersion in toluene on a 300 mesh Cu lacey carbon grid. Surface of HRTEM analyzed SiQDs was dodecyl terminated using the thermal hydrosilylation method. In the HRTEM images, nanoparticles fully fitting the image with clearly visible crystalline lattice were included in the analysis. Circles were manually drawn around the nanoparticles in ImageJ and the areas of the circles was calculated. The diameters were calculated based on the area of the drawn circles. We confirmed that this manual approach is independent of the individual executing it by having two different people analyze the same image containing about 20 nanoparticles. The independent analyses led to the same distribution of sizes, therefore, the error in drawing circles around well-defined nanoparticles is small enough not to influence the determined distribution of sizes.

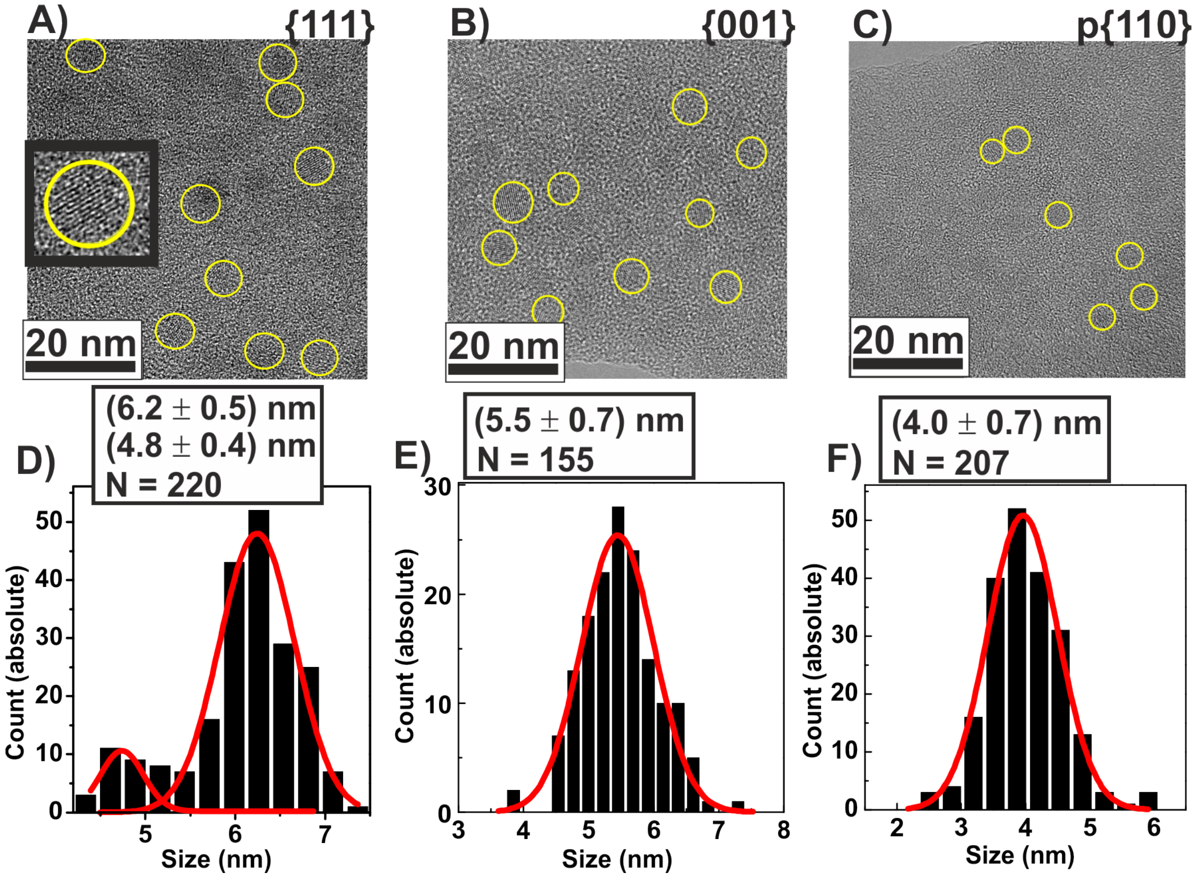

Figure S6: HRTEM characterization of SiQDs under study: (A–C) representative examples of the measurements with an enlarged QD shown in panel (A), (D–F) the corresponding histograms. The labelling is explained in the main text (Table 1). Mean sizes deduced from a statistically significant number of QDs together with the number of analyzed QDs are listed above the histograms, $^{\mathrm{FWHM}}/_{2}$ is used as the error to characterize the width of the distribution. Please note that the size distribution of the {111} sample is likely bimodal, the fraction of the smaller QDs is around 15 %.

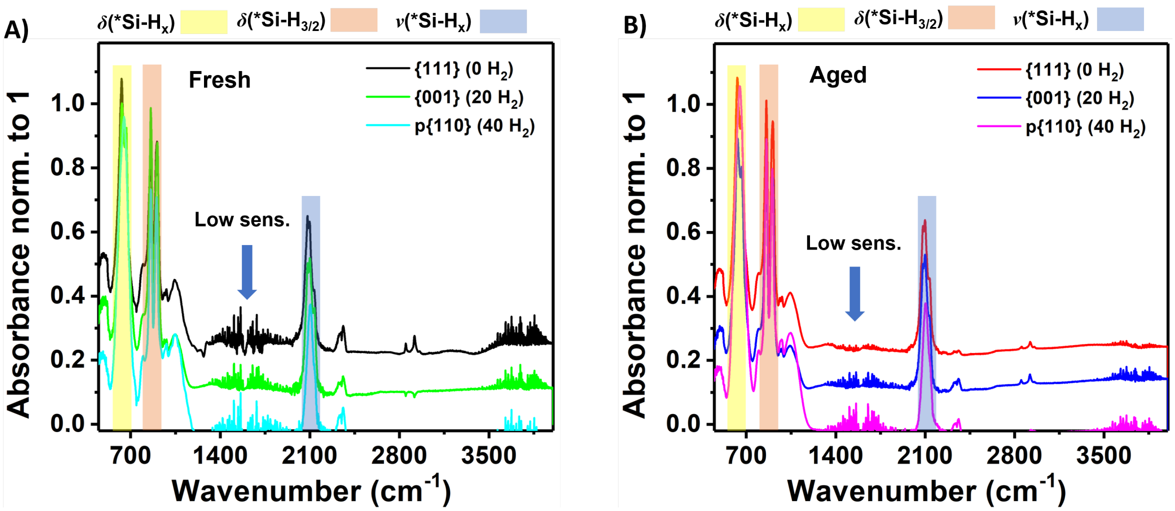

Figure S7: Full FTIR spectra of SiQDs under study, the labelling is explained in the main text (Table 1). The blue arrow marks the spectral region with low sensitivity, which causes higher levels of noise.

The Fourier-transform infrared (FTIR) absorption analysis was realized using Nicolet iS50 FT-IR microscope (Thermo Scientific). The measurements were performed in the attenuated total reflectance regime using a monocrystalline diamond crystal within the spectral range from 400 to 4000 cm^{-1} and the resolution of 4 cm^{-1} (256 accumulations). The Raman spectra measurements were realized using microspectrometer Renishaw inVia Reflex equipped with a HeCd continuous laser (Kimmon Dual Wavelength HeCd). The samples were characterized in the dry powder form using the excitation wavelength of 442 nm and low excitation power of 0.33 mW to avoid possible photoinduced modification of the sample. We cannot rule out that some of the observed shifts in the 520-cm^{-1} is caused by sample heating.

2.4 Determination of the ratios of surface hydrides

Based on published analysis,[S5] we used one peak for the SiH_3 surface stretching mode (2140 cm^{-1}), two peak for the stretching modes of SiH_2 (2100 and 2117 cm^{-1}) and two peak for the stretching modes of SiH (2070 and 2085 cm^{-1}). Therefore, the FTIR spectra were deconvoluted into five peaks corresponding with the vibrational modes, using the Renishaw WiRe software's curve fitting feature, which is built in the Renishaw software. The output was the peak position, integrated area, width and type of the spectral peak shape. We used the ratio of the integrated peaks to determine ratios of surface hydrides. Each datapoint of SiH_x composition represents at least six independent measurements.

2.5 Classical forcefield simulations of strain

The classical forcefield simulations were conducted with homemade code FireCore[S6] using harmonic spring $E = (K/2)(l - L)^2$ for description of bonds, cosine of half angle $E = A\cos((\theta - \theta_0)/2)$ for the angular terms and Lenard-Jones potential for non-covalent

interaction responsible for steric repulsion of the surface groups. The bonding parameters obtained from Ref. (S7) were $l_{Si-Si} = 2.37$ Å, $K_{Si-Si} = 9.487$ eV/Å^2 for Si–Si bond, $l_{Si-H} = 1.46$ Å, $K_{Si-H} = 15.604$ eV/Å^2 for the Si–H bond, $\theta_0 = 109.5°$ for all angles, and $A_{Si-Si-Si} = 5.667$, $A_{Si-Si-H} = 8.008$, $A_{H-Si-H} = 11.396$ eV/rad^2 for the angles. The Lenard-Jones force-field had parameters $R_{Si-Si} = 2.15$ Å, $E_{Si-Si} = 17.4321$ meV, $R_{H-H} = 1.40$ Åand $E_{H-H} = 0.9450$ meV and was using the Lorentz–Berthelot mixing rule for heterogeneous pairs of atoms. Note that our simulations is rather qualitative, but minor changes of the forcefield parameters are not expected to change the presented qualitative results.

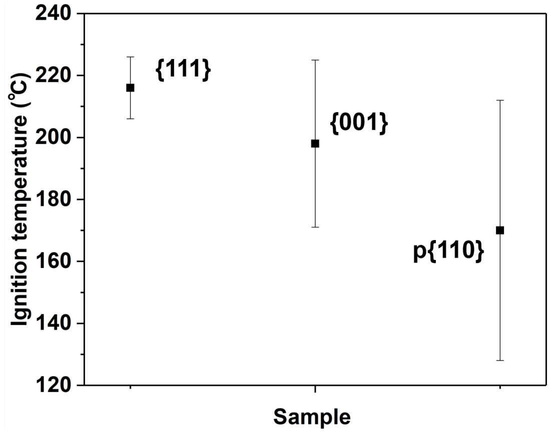

Figure S8: The ignition conditions of SiQDs from Table 1 in the main text. The samples were ignited on a hot plate.

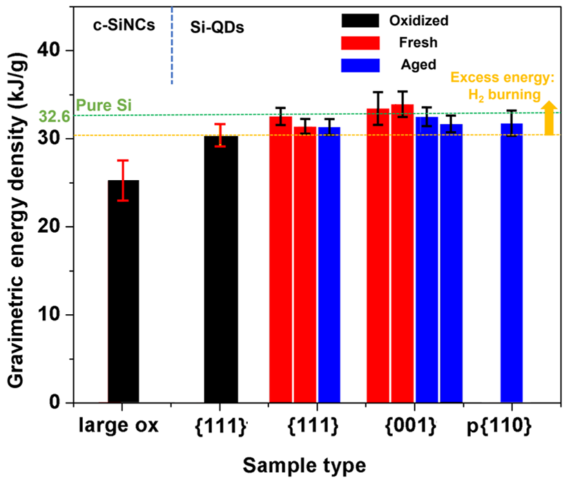

Figure S9: Calorimetric measurements of the set of SiQD samples from Table 1 complemented by calorimetric measurements of commercially available large silicon nanocrystals (170 nm, "large ox"). The error bars correspond to combined standard uncertainty. The p{110}-fresh SiQD sample burnt when it was introduced to the calorimetric chamber and thus could not be characterized.

2.6 Calorimetry

The gravimetric energy density was determined using a commercial Combustion compensated jacket calorimeter Parr, Model 1351, (Parr Instr. Comp., Moline, IL, USA), equipped with an oxygen bomb Parr 1108. The energy equivalent of the calorimeter was determined using benzoic acid (Parr instr., No 3415) as the calorimetric standard with declared heat of combustion $\Delta_c u^\circ = -6318$ I.T. cal/g, i.e. -26434.5 J/g, under certificate conditions. From six calibration experiments, the average energy equivalent value was $\eta_{\text{calor}} = (10066 \pm 3)$ J/K. From the separate experiments, the heat of combustion of a cotton thread (used for sample preparation and for sample ignition) with empirical formula $CH_{1.774}O_{0.867}$ was determined to be $\Delta_c u^\circ = (-16600 \pm 20)$ J/g, as well as the electric ignition heat (the permanent heat wire Parr Instr., No 840DD2 was used) $\Delta U_{\text{ign}} = 224$ J. Crepe paper with determined heat of combustion $\Delta_c u^\circ = (-16380 \pm 30)$ J/g was used for sample preparation, because the powder substance cannot be pressed into pellets. A volume of 1 ml of water was added into the bomb before each measurement, the oxygen pressure was $p = 3.04$ MPa. The sample gravimetric energy density values were calculated by the simplified procedure: only corrections to cotton thread, crepe paper and ignition heat were taken into account, the effect of the possible HNO_3 was neglected. The measurements uncertainty was calculated using Combined standard uncertainty combining standard deviating and precision of the measurements (sample/paper/cotton thread weighing, heat determination etc.).

2.7 DSC/TGA/MS

All samples were measured by non-isothermal DSC-TG analysis using the Setaram Themys 2400 simultaneous thermal analyzer, which was coupled by a quartz capillary with the Pfeiffer Vacuum OmniStarTM GSD320 mass spectrometer. Simultaneous DSC-TG-MS measurements of ca. $2 - 5$ mg of samples were performed in an alumina (Al_2O_3) crucible, in the Ar atmosphere (20 ml/min, purity 5.0N) and $Ar{+}O_2$ (15.9 ml/min Ar and 4 ml/min O_2 both of 5.0N purity and mixed in ratio 4:1), in a temperature range of $25 - 1100/1350$ ℃, with the heating range of 10 $K{\cdot}min^{-1}$. An empty alumina crucible was used as a reference. The blank run (baseline) was obtained before each thermoanalytical measurement of sample. The DSC was calibrated by Sn, Al, Ag, Ag_2SO_4, Au, and TG by $CuSO_4{\cdot}5H_2O$ and $CaC_2O_4{\cdot}H_2O$. The MS measurement was running in MID mode ("multiple ion detection") with the gas flow rate of $1 - 2$ sccm (at 0 ℃), using a secondary electron multiplier (SEM) detector operating at the voltage of $850 - 1450$ V, with dwell at each mass for 50 ms, and a resolution of 50, in the range of $1 - 50$ amu, and with a detection sensitivity for m/z signals smaller than 1 ppm. Data from all performed analyses (DSC-TGA-MS) were processed by Calisto Processing software. Each experiment was repeated $2 - 3\times$ for every sample, the repeated experiments led to very similar trends.

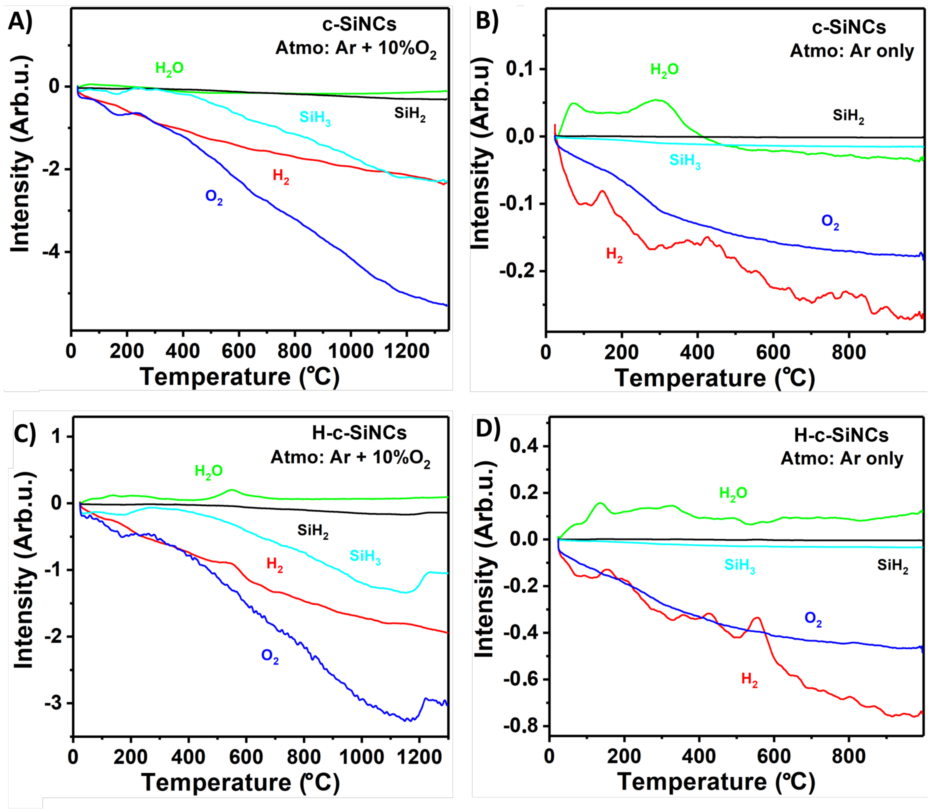

Figure S10: Mass spectroscopy analysis of C-SiNCs under oxidizing (a) and inert (b) atmosphere. Analogical measurements realized for hydrogen-terminated C-SiNCs are presented in panels (c) and (d), respectively.

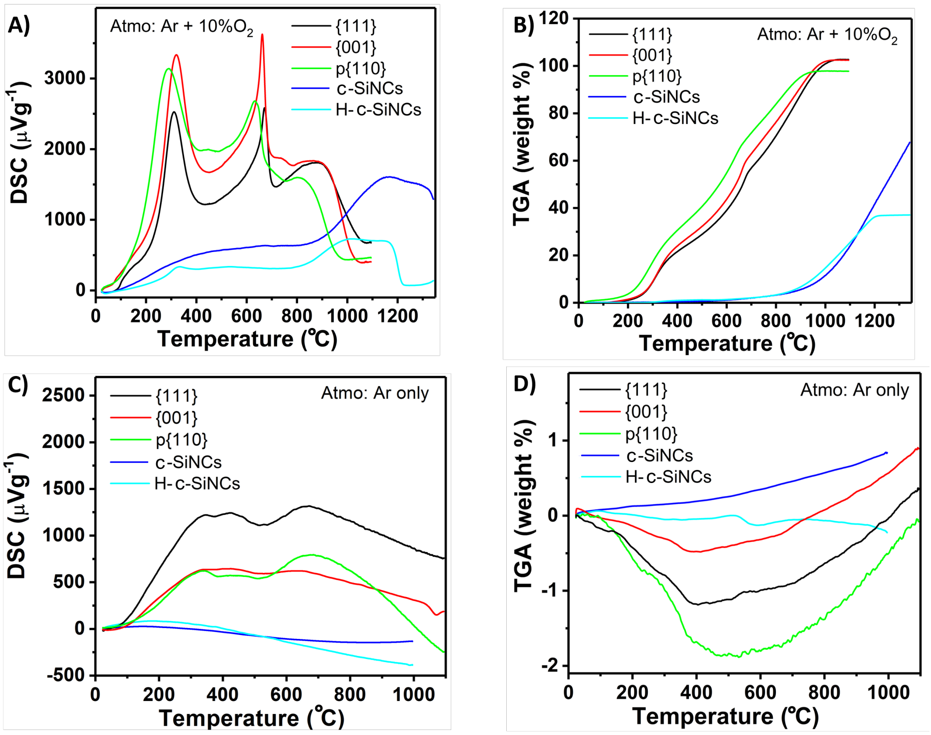

Figure S11: The comparison of DSC and TGA curves of indicated samples in the temperature interval 25 – 1100 ℃ under oxidizing and inert atmospheres.

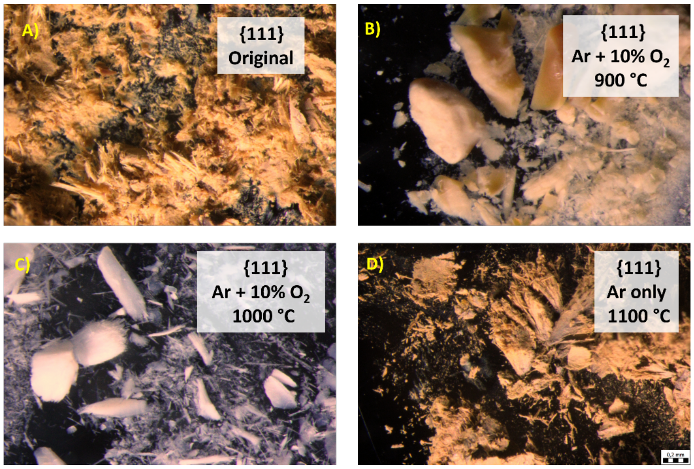

Figure S12: Ambient-lightning photos of the SiQD-{111} at various stages of the heating process as noted in the individual panels. The change of the color from beige-orange to white is indicative of full oxidation.

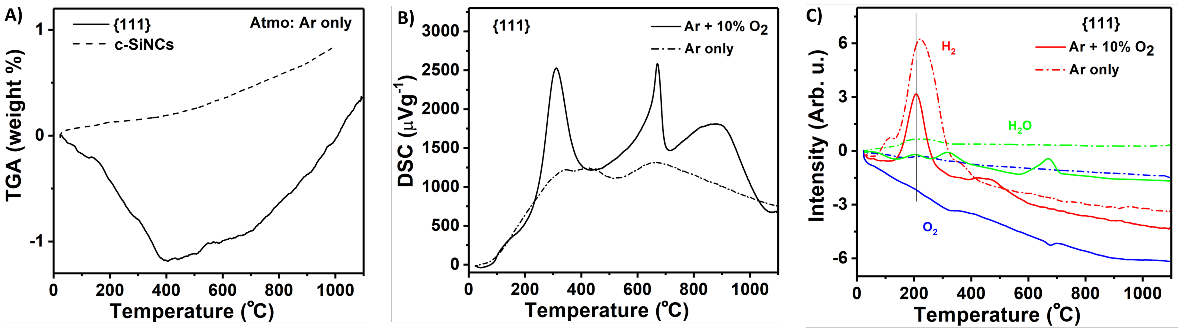

Figure S13: (a) TGA and (b) DSC measurements of SiQD-{111} and (oxidized) c-SiNCs under inert atmosphere. C) Comparison of MS of SiQD-{111} under oxidizing and inert atmosphere.

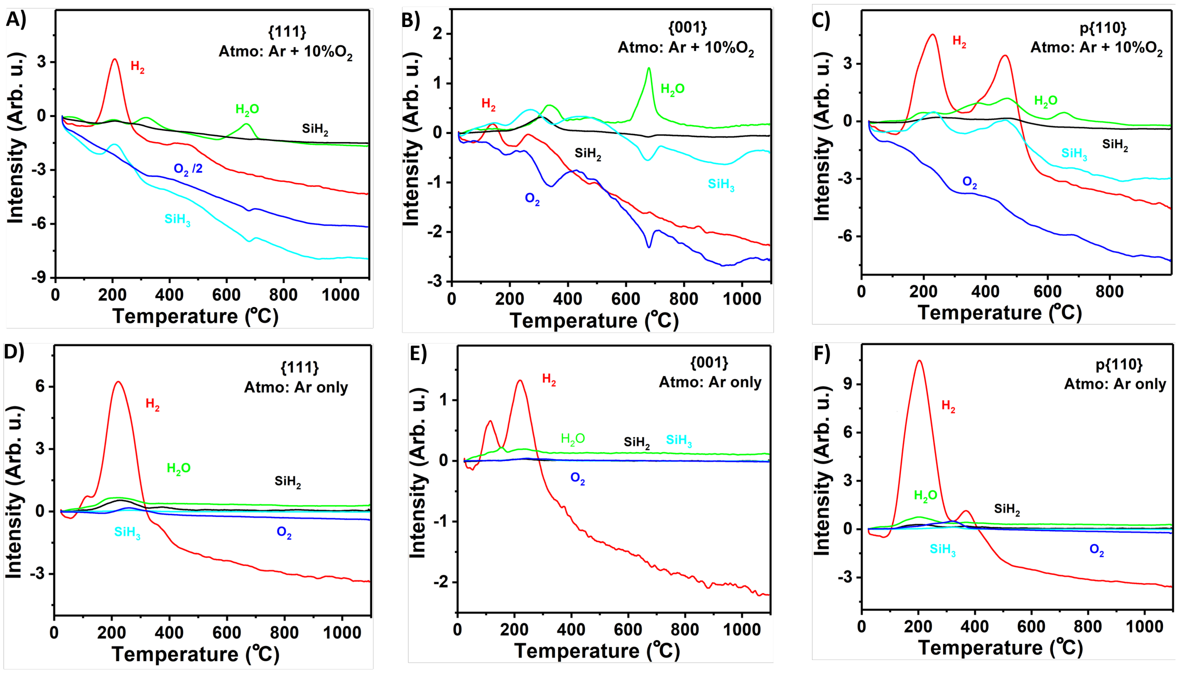

Figure S14: MS curves of fragments released during DSC/TGA/MS measurements. Type of atmosphere and sample is noted at each panel.

References

(S1) König, D. Number series of atoms, interatomic bonds and interface bonds defining zinc-blende nanocrystals as function of size, shape and surface orientation: Analytic tools to interpret solid state spectroscopy data. *AIP Adv.* **2016**, *6*, 085306.

(S2) Zeidler, E.; Hackbusch, W.; Schwarz, H. R. *Oxford User's Guide to Mathematics. Translated from German by Bruce Hunt.*; Oxford University Press, 2004.

(S3) Eaglesham, D. J.; White, A. E.; Feldman, L. C.; Moriya, N.; Jacobson, D. C. Equilibrium shape of Si. *Phys. Rev. Lett.* **1993**, *70*, 1643–1646.

(S4) Kortshagen, U. R.; Sankaran, R. M.; Pereira, R. N.; Girshick, S. L.; Wu, J. J.; Aydil, E. S. Nonthermal Plasma Synthesis of Nanocrystals: Fundamental Principles, Materials, and Applications. *Chem. Rev.* **2016**, *116*, 11061–11127.

(S5) Jariwala, B. N.; Kramer, N. J.; Petcu, M. C.; Bobela, D. C.; Sanden, M. C. M. v. d.; Stradins, P.; Ciobanu, C. V.; Agarwal, S. Surface Hydride Composition of Plasma-Synthesized Si Nanoparticles. *J. Phys. Chem. C* **2011**, *115*, 20375–20379.

(S6) Prokop, P.; Kočí, M.; Nicolini, P. ProkopHapala/FireCore. `https://github.com/ProkopHapala/FireCore`, 2024.

(S7) Pollard, W. B.; Lucovsky, G. Phonons in polysilane alloys. *Phys. Rev. B* **1982**, *26*, 3172–3180.
